# Susceptibility of the Placenta and Fetal Brain to Maternal Probiotic Supplementation

**DOI:** 10.3390/microorganisms14061175

**Published:** 2026-05-22

**Authors:** Rosalind T. B. Herrington, Zhen Lyu, David T. Ellenberger, Nathan J. Bivens, Zhentian Lei, Tanhaul Islam, Lloyd W. Sumner, R. Michael Roberts, Trupti Joshi, Cheryl S. Rosenfeld

**Affiliations:** 1 Pathobiology and Integrative Biomedical Sciences, University of Missouri, Columbia, MO 65211, USA; grh6mm@missouri.edu (R.T.B.H.); dtewfg@health.missouri.edu (D.T.E.); 2 Biological Sciences, University of Missouri, Columbia, MO 65211, USA; 3 Department of Biomedical Sciences, Joan C. Edwards School of Medicine, Marshall University, Huntington, WV 25703, USA; lyuz@marshall.edu; 4 Christopher S. Bond Life Science Center, University of Missouri, Columbia, MO 65211, USA; 5 Genomics Technology Core Facility, University of Missouri, Columbia, MO 65211, USA; bivensn@missouri.edu; 6 University of Missouri Metabolomics Center, University of Missouri, Columbia, MO 65211, USA; leiz@missouri.edu (Z.L.); tifd3@missouri.edu (T.I.); sumnerlw@missouri.edu (L.W.S.); 7 Biochemistry, University of Missouri, Columbia, MO 65211, USA; robertsrm@missouri.edu; 8 Animal Sciences, University of Missouri, Columbia, MO 65211, USA; 9 Department of Electrical Engineering and Computer Science, University of Missouri, Columbia, MO 65201, USA; 10 MU Institute for Data Science and Informatics, University of Missouri, Columbia, MO 65211, USA; 11 Department of Genetics Area Program, University of Missouri, Columbia, MO 65211, USA; 12 Thompson Center for Autism and Neurobehavioral Disorders, University of Missouri, Columbia, MO 65211, USA

**Keywords:** trophoblast, pregnancy, gut microbiome, bacterial metabolites, transcriptomics, metabolomics, short-chain fatty acids, SCFA

## Abstract

Probiotic supplements are increasingly being touted to have health benefits for pregnant women consuming such supplements and their unborn offspring. The placenta is in direct communication with maternal blood, and bioactive agents can thus easily be transferred to this organ where they may influence gene expression by the different trophoblast (TB) cell lineages. The underlying hypothesis assessed herein is that maternal probiotic supplementation can influence the placenta and fetal brain. The composition of bacterial short-chain fatty acids (SCFAs) was examined in fecal boli of mouse dams on a maternal probiotic supplement relative to control dams. Further, SCFA and transcriptomic profiles were examined in placenta and fetal brain from conceptuses derived from dams on the probiotic supplement and conceptuses from control dams. While this treatment did not affect bacterial SCFAs, placenta and fetal brain changes were evident in male and female conceptuses carried by dams receiving probiotics relative to controls. For the placenta, females were more sensitive to maternal probiotic supplementation, whereas the opposite was the case for the fetal brain. *Slc6a4* showed increased expression in female placenta from probiotic-treated dams, which could enhance uptake of maternal 5-HT. Male placenta from probiotic-treated dams had dramatic reduction in *Hsd11b2* that may render them more vulnerable to maternal stress. In the fetal brain, maternal probiotic supplementation was associated with genes linked to forebrain development, suggesting this treatment might impact life-long neurobehavioral responses. Current studies suggest that maternal probiotic supplementation might lead to adverse changes in the placenta and fetal brain of their unborn children.

## 1. Introduction

Probiotics have gained currency in treating and preventing various ailments. Increasing numbers of pregnant women use such biological supplements, whether based on their own research or on the advice of their obstetricians. Current estimates of probiotic usage by pregnant women globally range from 1.3 to 13.7% [1]. Health benefits touted to pregnant women consuming such compounds include reduced incidences of gastrointestinal disorders, eczema, pre-term birth, and adverse pregnancy outcomes, in particular gestational diabetes (GDM) and depression/anxiety [2,3,4,5,6,7,8,9,10,11,12,13,14,15,16,17]. One study found that probiotic milk intake by women late in pregnancy lowers pre-eclampsia risk, whereas consumption during early pregnancy led to a lower risk of pre-term delivery [16]. Probiotics might seem like the panacea for various conditions that plague pregnant women, but it is important to consider the general make-up of these supplements and how they might also impact their unborn offspring.

The Food and Agriculture Organization (FAO) of the United Nations and the World Health Organization (WHO)’s definition of probiotics refers to any “*living microorganism that, when administered in adequate quantities, have a health benefit to the host*” [18]. Probiotics are available without a prescription in various forms that range from freeze-dried bacteria, tablets, and capsules to powders. The premise of such bioactive compounds is that they are enriched in beneficial gut bacteria, with most formulations consisting of two p bacterial genera: *Lactobacilli* spp. and *Bifidobacteria* spp. These bacteria are presumed to have a symbiotic relationship with the host gut, skin, vagina, and other organs. These regions provide a surface where such bacteria can presumably colonize and replicate. In return, such bacteria yield factors and hormones that promote our survival and health [19].

The placenta is in direct communication with the maternal blood and acts as a communication organ to allow for the transfer of maternal nutrients, gases, and fetal waste. The placenta is vulnerable to changes in in utero environmental conditions, including compounds consumed by the mother. Further, the placenta comprises diverse trophoblast (TB) cells that release a range of hormones and other compounds that govern normal maternal and fetal responses. Such biomolecules consist of neurotransmitters, such as serotonin (5-HT), catecholamines, miRNA, and other chemicals, which guide early fetal brain formation [20,21,22,23,24,25]. The tight dependency between these two organs has given rise to the term the placenta–brain axis [20,21,22]. Consequently, factors transferred from the maternal blood to the placenta, e.g., bacterial and maternal-derived nutrients, can impact this axis.

In a recent review, we explored potential mechanisms of how maternal probiotic supplementation might impact the placenta–brain axis [26]. Three potential mechanisms were considered. The first posits that such microorganisms might enter the maternal circulation and colonize the placenta, once thought to be a sterile organ, although there is extensive controversy on whether bacteria reside in a healthy placenta, as discussed in this recent review [26]. A second and more likely mechanism is that maternal supplementation with such microorganisms might alter maternal gut bacterial metabolites, including short-chain fatty acids (SCFAs) that can then circulate and impact the placenta and fetal brain. The last mechanism considered in this recent review was that maternal gut bacteria modulation might impact maternal cellular and humoral immunity, as well as immune responses within the placenta.

While there have been several studies examining how maternal probiotic supplementation during lactation/the perinatal period affects maternal behavior and offspring outcomes [27,28,29], a scant amount is known about how such treatment in pregnant mice impacts the conceptus. One study showed that providing pregnant mice with fermented milk containing *L. plantarum* IIA-1A5 did not alter fetal morphology or skeletal muscle development [30]. Another study that inoculated germ-free (GF) pregnant mice with *Bifidobacterium breve* UCC2003 reported that this treatment altered placental morphogenesis, nutrient transport and fetal growth [31]. At gestational day 16.5, the placenta weighed less in conceptuses carried by GF pregnant mice and GF pregnant mice inoculated with *Bifidobacterium breve* compared to placentas from specific pathogen-free (SPF) females [31]. The placental labyrinth zone showed reduced growth in GF females. The placenta of conceptuses derived from GF pregnant dams with this bacterium had metabolite changes in the placental junctional zone, upregulated expression of nutrient carriers, in particular *Slc7a8* and *Slc16a4*, and increased amounts of prolactins and pregnancy-specific glycoproteins relative to control GF dams. Angiogenesis and associated factors showed disruptions in the junctional and labyrinth zones for placentas derived from GF dams not treated with any probiotic [32]. Prolactin genes were downregulated in the placenta of conceptuses carried by pregnant untreated GF dams relative to the placenta of conceptuses derived from specific pathogen-free (SPF) pregnant females [33]. In pregnant women provided a probiotic supplement containing *Bifidobacterium lactis* alone or in combination with *Lactobacillus rhamnosus GG (LGG)*, these probiotic treatments altered the expression of toll-like receptor (TLR)-related genes both in the placenta and in the fetal gut (meconium) following cesarian section [34]. Maternal probiotic supplementation by women late in pregnancy has been associated with beneficial effects on infant weight and length gains when measured at 12 months of age [35].

In the current research, we focus on whether probiotic supplementation might alter SCFA in the maternal gut, as measured in maternal fecal boli, placenta, and fetal brain. Additionally, we determined whether maternal probiotic supplementation alters transcriptomic profiles in the placenta and fetal brain.

## 2. Materials and Methods

### 2.1. Probiotic Exposure

These mouse studies were approved by the University of Missouri Animal Care and Use Committee (ACUC, Protocol #45687). Eight adult (~8–12 week old) wild-type C57Bl6J mouse dams, which were purchased from Jackson Laboratory (Bar Harbor, ME, USA), were randomly assigned to receive one capsule of a probiotic supplement (Bio-Kult Everyday Probiotic Supplement, BIOKULT LIMITED, Manchester, UK) that was dispensed in 100 mL of water and was changed three times weekly or receive water alone (controls) for two weeks before pairing for breeding and continued to receive the probiotic throughout pairing and pregnancy. This randomization process involved assigning each of the mice a number and then randomly assigning them to one of the two research groups (probiotic treatment or vehicle control). This specific probiotic supplement was chosen as it has previously been shown to induce developmental origins of adult health and disease (DOHaD) effects, including neurobehavioral responses, when administered to pregnant mice [27].

After the mice were assigned, we determined the average and standard error of the mean (SEM) weight to ensure the two groups did not show any differences in weight at the outset of the studies. Mice were maintained on a 12:12 h dark:light cycle. Mice were provided standard Purina 5001 diet (Purina, St. Louis, MO, USA). The bedding was aspen shavings purchased from Bourn Feed (Columbia, MO, USA). Each female was provided a nestlet (Ancare Corp, Bellmore, NY, USA) to build a nest, and this provided environmental enrichment for the mice. Cages, aspen bedding, and nestlets were changed once weekly as per our animal institute requirements. The cages were also randomized on each shelf in the rack to avoid any positional bias. Females were housed singly to monitor individual water consumption. The probiotic was administered in the drinking water as this is a non-invasive exposure method that has been shown to lead to effective concentrations for altering the gut bacteria and correspondingly providing beneficial neuroprotective effects [27,36]. While oral gavage might ensure the animals receive the treatment, this method can be stressful to the mice, result in damage to the oral cavity/esophagus, and lead to confounding effects [37,38,39,40]. As determined previously based on a similar frequency of water changes per week [27], periconception and pregnant female mice assumingly received 4 × 10^7^ colony-forming units per milliliter or vehicle alone for those groups not receiving the probiotic supplement. Previous work suggests that this should correspond to an intake of ∼6.4 million live bacteria per gram of body weight per day, based on the average daily water consumption of an adult mouse (0.12 mL/g per mouse per day) [27]. Notably, this same probiotic supplement and treatment regimen increased the relative amounts of fecal *Lactobacillus* spp. and *Bifidobacterium* spp. relative to control females [27]. No differences in water consumption were detected in the previous study or in the current work, as detailed below. The bacteria within this supplement are listed in Table S1 (page 9 in Supplementary Package). In short, this probiotic supplement includes 1 species of Bacillus, 4 species of Bifidobacteria, 5 species of Lactobacillus, 1 species of Lactococcus, and 1 species of Streptococcus. Four female mice were assigned to each of the groups. This number of replicates was determined based on the aforementioned study that exposed female mice to the same probiotic and route of exposure [27]. We, and others, who have examined how maternal changes in mice can affect the placenta have primarily tested four replicates per group [24,31,41,42,43,44,45,46,47]. These reports include the study that examined placental changes following supplementation of the bacterium *Bifidobacterium breve* UCC2003 in GF mice [31]. For our previous studies and current work, this number was chosen based on a power analysis of 0.8. Males were paired each night with the females, and each morning, females were examined for a vaginal plug. Males were removed from the cages containing these females. If no vaginal plug was noted, the male was introduced that night, and females were plug-checked the next morning until all females had been bred. A summary of the experimental design is provided in Figure S1 (page 2 in Supplementary Package).

### 2.2. Collection of Maternal Fecal Boli Samples

Four to seven fecal boli were collected from each dam prior to being placed on probiotic supplement or vehicle control (pre-exposure) and just prior to euthanasia (post-exposure). Prior to fecal collection, dams were placed in clean and empty cages for up to fifteen minutes, and their fecal boli were collected.

### 2.3. Collection of Fetal Placenta and Brain Samples

Females from both groups who had been previously bred, as detailed above, were weighed daily and culled at 12.5 dpc by American Veterinary Medical Association (AVMA)-approved euthanasia methods (CO_2_ followed cervical dislocation). Fetal placentas and brains were frozen in liquid nitrogen. Fetal tissue was collected for embryonic sexing procedures. This period following breeding was chosen as it approximates mid-gestation when the placenta is fully mature. We, and others, have shown that this period is when the placenta is sensitive to in utero perturbations. This period also represents when the brain is initially forming and most sensitive to placental-derived factors, e.g., serotonin [20,22].

### 2.4. PCR Sexing

Sex analysis of embryonic tissues was performed by extracting DNA from fetal tissue with the Qiagen DNeasy Blood and Tissue DNA extraction kit (Cat. No. 69504; Qiagen, Germantown, MD, USA). Following DNA extraction, PCR analysis was conducted. To assess whether a conceptus was male or female, we conducted PCR based on the *Sry* gene. The forward primer used for the SRY gene was 5′TCATGAGACTGCCAACCACAG3′ and the reverse primer was 5′CATGACCACCACCACCACCAA3′. We also concurrently examined for expression of *Gapdh* to verify the absence of an *Sry* amplicon was not due to DNA degradation. The forward primer for the *Gapdh* gene was 5′ACCACAGTCCATGCCATCAC3′, and the reverse primer was 5′TCCACCACCCTGTTGCTGTA3′. All primers were supplied by Integrated DNA Technologies (IDT, Coralville, IA, USA). PCR analysis was done with the Green Master Mix (Promega, Madison, WI, USA) and according to the manufacturer’s recommendations. The reactions were run on 2% agarose (ThermoFisher Scientific, Waltham, MA, USA) gel containing ethidium bromide (ThermoFisher Scientific) with gel electrophoresis for one hour at 200 milliamps and 141 volts. Sex was determined based on the presence of a 440 bp amplicon for the *Sry* gene on the gel: samples lacking this product were classified as female, provided they had the correct amplicon size (434 base pairs) for *Gapdh*, while those with the expected product size were classified as male. To avoid overlapping the amplicon products, separate PCR reactions and electrophoresis gels were run for *Sry* and *Gapdh*.

### 2.5. Short-Chain Fatty Acid Analysis from Maternal Fecal Boli, Placenta, and Fetal Brain Samples

We chose to examine the SCFAs 2-methylbutanoic acid, isovaleric acid, valeric acid, Butyric acid, isobutyric acid, propanoic acid, and acetic acid as these SCFAs have previously been shown to induce placental alterations [26]. These include impacting trophoblast function, placental immune/inflammatory responses, and metabolic signaling in this organ, and epigenetic changes [26,48,49,50,51,52,53,54,55,56,57]. SCFAs influence the placental expression of tryptophan 5-hydroxylase 1, which is an enzyme regulating serotonin synthesis [58]. The mouse and human placenta appear to produce serotonin that regulates early forebrain development [20,22,59,60,61]. SCFAs stimulate proliferation of human neural progenitor cells [62]. Thus, SCFA changes in the dam might affect the placenta and early fetal brain.

To measure these SCFAs, samples (maternal fecal samples, placenta, and fetal brain) were weighed and mixed with 50% acetonitrile containing internal standard 2-isobutoxyacetic acid (15 μg/mL) at a ratio of 1:20 (*w*/*v*, g/mL). Four replicates for each sample and group were evaluated. The samples were homogenized by using BeadBeater. The homogenized samples were transferred into Eppendorf tubes and centrifuged at 13,000 *g* for 15 min. After centrifugation, 50 μL of supernatant was transferred into a glass sample vial insert for derivatization. Derivatization was performed by sequentially adding 10 μL of 100 mM of 3-NPH (3-Nitrophenylhydrazine), 10 μL of 100 mM of EDC (N-ethyl-N′-(3-dimethylaminopropyl)carbodiimide), and 10 μL of pyridine (15%) in methanol and incubating at 37 °C for 45 min.

LC-MS/MS MRM was performed in a positive ion mode on a Waters Xevo TQ XS triple quadrupole mass spectrometer coupled to a Waters UPLC system. Standards (acetic acid, propionic acid, butyric acid, isobutyric acid, valeric acid, isovaleric acid and 2-methylbutanoic acid at 4 μL/mL) were also derivatized in the same manner as a positive control. LC chromatographic separation was achieved on a C18 column (2.7 μm, 2.1 × 150 mm) using as mobile phase (A) water +0.1% formic acid and (B) acetonitrile. The elution program (% B) was 0–2.5 min 10%, 2.5–16 min 10–50%, 16–18 min 50%, and 18–18.2 50–10% maintained for 20 min. The flow rate was 0.4 mL/min, and the column and the autosampler temperature were 35 °C and 10 °C, respectively. Two MRM transitions monitored for each short-chain fatty acid are shown in Table S2 (page 10 in Supplementary Package).

Standard calibration curves for each SCFA were generated by using a standard dilution series and used to quantify SCFAs in the samples. The principal component analysis (PCA) plot was generated with MetaboAnalyst software (Version 6.0, https://www.metaboanalyst.ca/faces/ModuleView.xhtml, accessed on 16 March 2026).

### 2.6. Statistical Analyses

For the maternal body weight, water consumption data, maternal fecal SCFA concentrations, placental SCFA concentrations, and fetal brain SCFA concentrations, the mean and standard error of the mean (SEM) of the sample average was calculated for each group. Statistical significance was assessed by using an unpaired *t*-test (GraphPad, Version 10.5.0, San Diego, CA, USA), and graphs were created with Microsoft Excel (version 16.99.2). Statistically significant samples had a *p* value ≤ 0.05.

### 2.7. RNA Isolation from Fetal Placenta and Brain Samples

To characterize changes in gene expression patterns in control and probiotic conceptuses, total RNA was extracted by using the Qiagen AllPrep DNA/RNA/miRNA Universal Kit (Cat. No. 80224; Qiagen, MD, USA) in accordance with the manufacturer’s protocol. RNA was extracted and analyzed from four male experimental placenta, four female experimental placenta, four male control placenta, four female control placenta, four male experimental fetal brains, three female experimental fetal brains, four male control fetal brains, and four female control fetal brain tissues. RNA concentration was initially assessed with a NanoDrop ND-1000 spectrophotometer (NanoDrop Products, Wilmington, DE, USA) and further evaluated for integrity with the Fragment Analyzer system (Agilent Technologies Inc., Santa Clara, CA, USA). Only those samples that had an RNA integrity number (RIN) score ≥ 7.0 were selected for RNA sequencing (RNAseq).

### 2.8. Illumina TruSeq RNA Library Preparation and Sequencing

Libraries were constructed following the manufacturer’s protocol with reagents supplied in the Illumina mRNA Stranded Library Preparation Kit and sequenced at the University of Missouri Genomics Technology Core. In brief, mRNA was captured with oligo(dT) magnetic beads, eluted, fragmented and then primed for cDNA synthesis. cDNA was synthesized using hexamer-primed RNA fragments and reverse transcriptase. After completion of second-strand cDNA synthesis pre-index anchors were ligated to the ends of the cDNA. A subsequent PCR step was used to selectively amplify the anchor-ligated DNA fragments and add indexes and primer sequences for cluster generation. The final amplified cDNA constructs were purified by addition of Axyprep Mag PCR Clean-up beads (Fisher Scientific).

The final library size was evaluated on a Fragment Analyzer (Agilent Technologies, Inc., Santa Clara, CA, USA), quantified with the Qubit fluorometer by means of the Qubit dsDNA HS Assay kit (Thermo Fisher Scientific, Waltham, MA, USA), and diluted according to Illumina’s standard sequencing protocol. Libraries were pooled and run on an Illumina NovaSeq X sequencer with a paired end 100 bp read format on a 10B flow cell to generate ~100 million paired reads per sample.

### 2.9. RNAseq Data Processing

The RNA-seq data analysis pipeline was initiated with a quality assessment of the raw Illumina sequencing reads by using FastQC (version 0.12.1) [63]. High-quality reads were then aligned to the mouse reference genome (GRCm39) by using the Hisat2 (version 2.1.0) aligner with its index-building functionality [64] (Table S3, page 11 in Supplementary Package). Following alignment, transcript assembly and quantification were conducted by using Cufflinks (version 2.2.1) program [65], which estimates gene expression levels in terms of FPKM (Fragments Per Kilobase of transcript per Million mapped reads). For RNAseq analyses, three replicates per group is considered acceptable, especially if there is in-depth sequence coverage [66]. As shown in Table S3, the average raw reads were 155099206.8, mapped reads were 152322686.4, and percentage of mapped reads was 98.2%. These numbers are more than sufficient for an eukaryote genome [67]. To identify differentially expressed genes (DEGs), we used Cuffdiff (version 2.2.1). Cufflinks/Cuffdiff were chosen because these platforms integrate seamlessly with our transcript assembly workflow, enable direct estimation of transcript-level expression and isoform variation, and have been successfully applied in prior studies with similar datasets and objectives [68]. For both placenta and brain samples, DEGs were defined by using a log_2_ fold change (log2FC) threshold of 1 and a q-value < 0.05.

Visualization of differential expression results was performed by using Python version 3.10.18. Volcano plots were generated for each comparison using the matplotlib package (version 3.10.0) [69] to highlight significantly upregulated and downregulated genes. Principal component analysis (PCA) was conducted on normalized gene expression counts using the scikit-learn library (version 1.6.1) [70] to assess sample clustering, and the resulting components were visualized with the seaborn package.

### 2.10. Enrichment and Network Analyses

Enrichment analyses were conducted by using TissueEnrich [59], the mouse ENCODE dataset [71] and EnrichR [72] with the KEGG_2019_Mouse database. For functional enrichment analysis, DEGs were imported into the WEB-based GEne SeT AnaLysis Toolkit (WebGestalt) 2019 version and searched for gene ontology molecular function (GO MF) and gene ontology biological processes (GO BP) [70].

### 2.11. Correlation Analyses of Metabolomics and Transcriptomics Data

For these correlation analyses, comparisons were performed separately for each RNA-seq group. Differentially expressed genes (DEGs) identified from each comparison were selected and correlated with metabolomics data by using R. Correlation analyses were conducted based on Kendall’s rank correlation coefficient to assess monotonic associations between gene expression levels and metabolite abundances. The results were visualized by using the corrplot package (version 0.95) [73]. Statistically significant correlations (*p* < 0.05) are highlighted with a red rectangle surrounding the correlation plot to emphasize meaningful transcript–metabolite associations.

### 2.12. Data Availability

RNA sequencing data have been deposited in the Gene Expression Omnibus under accession: GSE309308 (https://www.ncbi.nlm.nih.gov/geo/query/acc.cgi?acc=GSE309308, accessed on 16 March 2026).

## 3. Results

### 3.1. Maternal Body Weight and Water Consumption

Body weights for females prior to being placed on probiotic supplement or vehicle were not significantly different (19.62 ± 0.62 g vs. 20.37 ± 1.14 g, respectively). However, pregnant mice females weighed more at time of euthanasia than control dams (33.873 ± 0.47 g vs. 31.12 ± 0.51 g, respectively, *p* = 0.008, Figure S2A, page 3 in Supplementary Package). Water consumption did not differ for probiotic vs. control dams when measured every 48 h, whereupon new water containing vehicle control or probiotic supplement was provided (17.46 ± 0.94 mL vs. 15.82 ± 1.19 mL, respectively, Figure S2B, page 3 in Supplementary Package). Litter size did not differ between controls or dams on the probiotic treatment (8 ± 0.67 vs. 8 ± 0.58, Figure S2C, page 3 in Supplementary Package).

### 3.2. Short-Chain Fatty Acids (SCFAs) Within Maternal Fecal Samples

PCA for all the SCFAs examined and three groups—(1) female mice prior to probiotic treatment, (2) control female mice prior to exposure to vehicle control, (3) female mice following two weeks exposure to probiotic supplement—during the peri-conception period and pregnancy up until when conceptuses were collected did not show clear separation based on group (Figure S3, page 4 in Supplementary Package). As shown in Table S4 (page 13 in Supplementary Package) none of the SCFAs differed in the fecal samples following maternal treatment with the probiotic supplement. In all fecal boli samples, acetic acid was the most common SCFA followed by propanoic acid and butyric acid.

### 3.3. Short-Chain Fatty Acids (SCFAs) Within Placenta and Fetal Brain Samples

**Figure 1:**
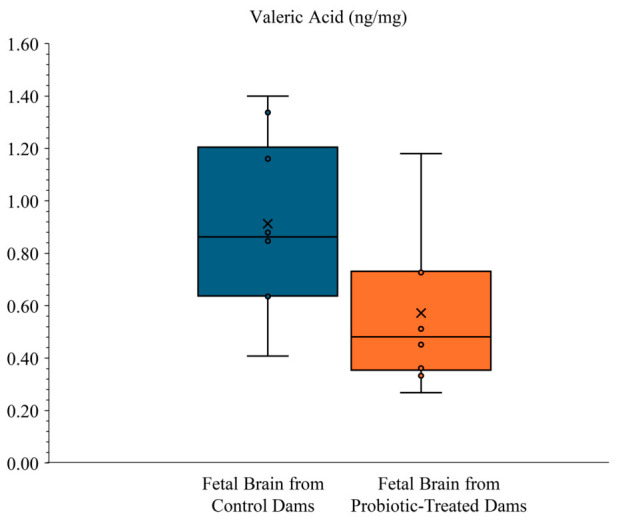
Concentrations of valeric acid in fetal brain samples from conceptuses derived from control vs. probiotic-treated dams. N = 4 replicates evaluated per group. The open circles represent individual data points. The X mark represents the mean value for each group. The horizontal line within the bar graph represents the median value.

As no differences were detected based on conceptus sex, male and female data were combined for placenta and for fetal brain samples. This approach has been used previously in cases where no sex differences are noted [74]. Figure S4 (page 5 in Supplementary Package) shows the PCA plot based on all SCFAs for placenta and fetal brain from the two maternal treatment groups: probiotic supplement and vehicle control. The only SCFA that differed based on maternal treatment was valeric acid, which was lower in fetal brain samples in those dams who received the probiotic supplement compared to control dams (fetal brain from control dams: 0.91 ± 0.13 ng/mg tissue; vs. 0.57 ± 0.11 ng/mg, *p* = 0.03, Figure 1). Results for all other SCFAs in placenta and fetal brain samples from these two maternal treatment groups are listed in Table S5 (page 14 in Supplementary Package). As shown, acetic acid, followed by propanoic acid, is the most abundant SCFA in placenta and fetal brain samples for both treatment groups.

While the SCFAs did not show striking differences other than valeric acid, maternal probiotic supplementation might affect the placenta and brain through alteration of other metabolites and via other mechanisms, as detailed above. Thus, we sought next to determine if this treatment altered the transcriptomic profile in the placenta and fetal brain.

### 3.4. RNA Sequencing

#### 3.4.1. General Features Based on Organ, Maternal Treatment, and Sex Differences

The average number of reads for all samples was 155,099,206.8, the average number of mapped reads was 152,322,686.4, and the average percentage of alignment was 98.21% (Table S3, page 11 in Supplementary Package). No clear separation was evident based on maternal treatment (probiotic vs. control) or sex (Figure S5, page 6 in Supplementary Package). PERMANOVA values for placenta and fetal brain were 0.66 and 0.54, respectively. However, volcano plot analyses revealed that at q value ≤ 0.05 and 2-fold change difference, 211 transcripts differed between maternal probiotic female placenta samples vs. control female placenta with 194 upregulated in probiotic female placenta samples and 17 upregulated in control female placenta samples (Figure S6A, page 7 in Supplementary Package). For male placenta samples, 63 differentially expressed genes were identified between probiotic vs. control with 52 upregulated in probiotic and 11 upregulated in control placenta (Figure S6B, page 7 in Supplementary Package). For brain samples, 55 transcripts were differentially expressed for female brain samples with 26 upregulated in probiotic and 29 upregulated in controls (Figure S6C, page 7 in Supplementary Package). For male brain samples, 207 showed differential expression based on these analyses, with 100 upregulated in probiotic and 107 upregulated in controls (Figure S6D, page 7 in Supplementary Package).

#### 3.4.2. Differentially Expressed Genes in Placenta Based on Maternal Treatment and Sex Differences

**Figure 2:**
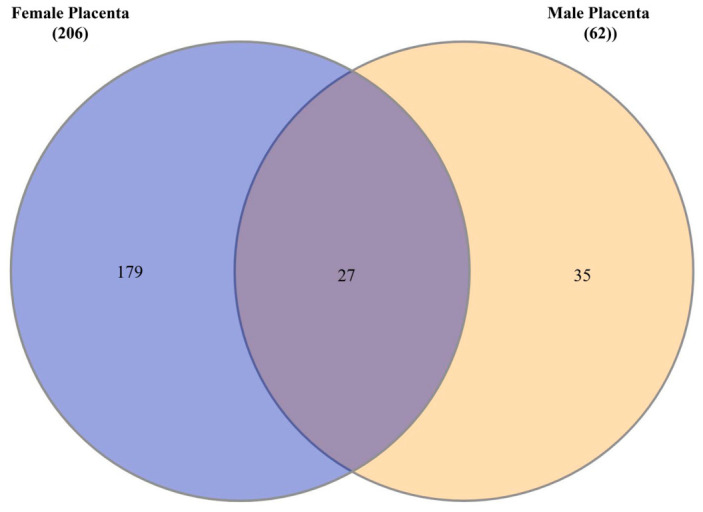
Venn diagram gene expression changes in placenta from female and male conceptuses for probiotic-treated vs. control dams. As shown, the placenta of female conceptuses demonstrated a greater number of gene expression changes in response to maternal probiotic supplementation compared to male siblings. N = 4 replicates evaluated per group.

Of the 206 known differentially expressed transcripts in female placenta compared to the 62 known differentially expressed genes in male placenta, 27 genes overlapped when comparing probiotic vs. control placental samples (Figure 2). The female comparison had 179 that were unique, and 35 transcripts were only differentially expressed for male comparison. These are listed in Supplementary File S1 with overlap genes shown in column B of this file. The full list of differentially expressed genes for female placenta is provided in Supplementary File S2, and those for male placenta is provided in Supplementary File S3. The most up- and downregulated transcripts based on fold change for female placenta are shown in Table 1 and those for male placenta are listed in Table 2. These tables also include general functions for these genes.

### 3.5. Target Enrichment and Predicted Diseases/Pathways in Placenta Based on Maternal Treatment and Sex Differences

**Figure 3:**
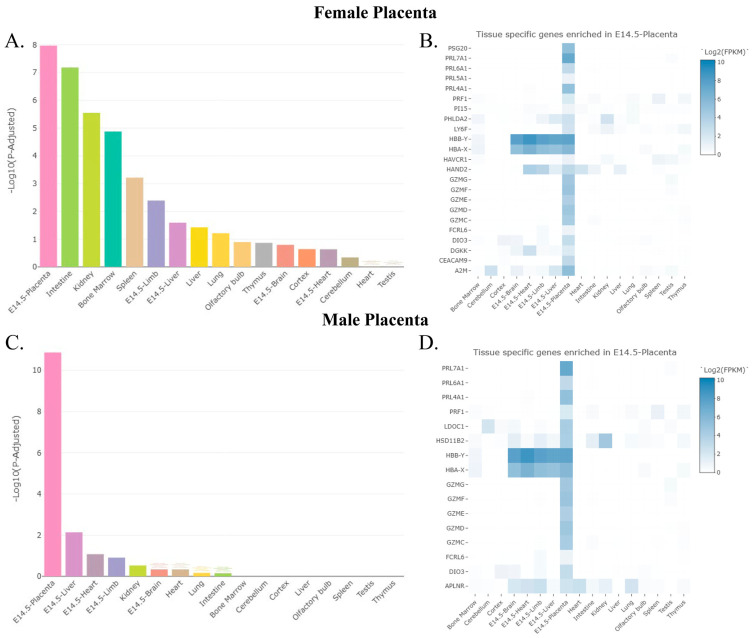
Tissue enrichment and heatmap analyses for genes that show differential expression in female and male placenta from probiotic-treated vs. control dams. (**A**) Tissue enrichment for differentially expressed genes (DEGs) in female placenta for this comparison. (**B**) Heat map analysis of genes enriched in the placenta based on DEGs in female placenta of probiotic-treated vs. control dams. (**C**) Tissue enrichment for DEGs in male placenta of probiotic-treated vs. control dams. (**D**) Heat map analysis of genes enriched in the placenta based on DEGs in male placenta of probiotic-treated vs. control dams.

The TissueEnrich program [75] was then used to determine which organs of the mouse have an abundance of transcripts for differentially expressed genes in female and male placenta, and in the case of the placenta, those transcripts that might be exclusively expressed in this organ. For the female placenta, the differentially expressed transcripts are primarily expressed in the placenta followed by the intestine, kidney, bone marrow, spleen, embryonic limb, and liver (Figure 3A). The heat map generated from this program reveals that many of the differentially expressed transcripts that are enriched in the placenta include several prolactin forms, including *Prl7a1*, *Prl6a1*, *Prl5a1*, *Prl5a1*, and *Prf*, as well as *Hand2* and *Ceacam9* (Figure 3B). For male placenta, the differentially expressed transcripts are primarily associated with the placenta, liver, heart, limb, and kidney (Figure 3C). The heat map generated by this program reveals that many of the differentially expressed transcripts that are enriched in the placenta also include several prolactin forms, including *Prl7a1*, *Prl6a1*, *Prl4a1*, and *Prf*, as well as *Hsd11b2* and *Dio3* (Figure 3D).

**Figure 4:**
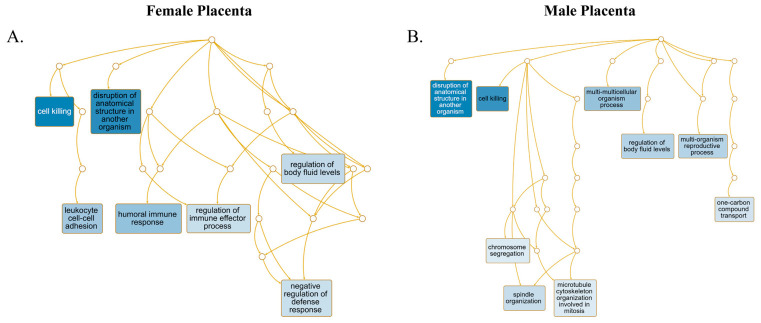
Pathways associated with DEGs in female and male placenta for probiotic-treated vs. control dams. (**A**) Pathways associated with DEGs for female placenta. (**B**) Pathways associated with DEGs in male placenta. The color of the box around each pathway indicates statistical significance with light blue indicating a *p* value < 0.05, and dark blue indicating significance at a q value (FDR) <0.05.

Pathways associated with differentially expressed transcripts in female placenta include those associated with cell killing, anatomical disruption, leukocyte cell–cell adhesion, humoral immune response, regulation of the immune effector process, regulation of body fluid levels, and negative regulation of the defense response (Figure 4A). In contrast, those associated with genes differentially expressed in male placenta include those linked with anatomical disruption, cell killing, chromosome segregation, spindle organization, microtube cytoskeleton organization associated with mitosis, regulation of body fluid levels, reproductive processes, and one carbon compound transport (Figure 4B).

### 3.6. Differentially Expressed Genes in Brain Based on Maternal Treatment and Sex Differences

**Figure 5:**
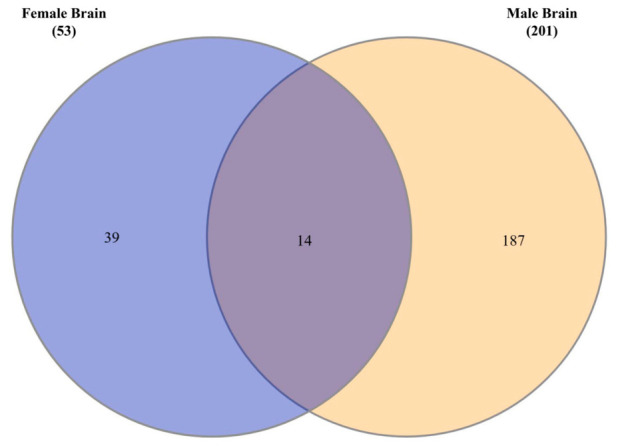
Venn diagram gene expression changes in fetal brain from female and male conceptuses for probiotic-treated vs. control dams. As shown, the brains of male conceptuses demonstrated a greater number of gene expression changes in response to maternal probiotic supplementation compared to female siblings. N = 4 replicates assessed per group.

A Venn diagram comparison reveals that of the 53 differentially expressed and known transcripts in female brain compared to the 201 differentially expressed and known genes in male brain, 14 genes overlapped (Figure 5). The female comparison had 39 that were unique, and 187 transcripts were only differentially expressed for male comparison. These are listed in Supplementary File S4 with overlap genes shown in column B of this file.

The full list of DEGs based on *p* value only for female fetal brain derived from conceptuses for probiotic-treated dams compared to female fetal brain derived from conceptuses for control dams is provided in Supplementary File S5, and those for male fetal brain derived from conceptuses for probiotic-treated dams compared to male fetal brain derived from conceptuses for control dams is provided in Supplementary File S6. The most up- and downregulated transcripts based on fold change for female fetal brain are shown in Table 3 and those for male fetal brain are listed in Table 4. These tables again include general functions for these genes.

### 3.7. Target Enrichment and Predicted Diseases/Pathways in Brain Based on Maternal Treatment and Sex Differences

**Figure 6:**
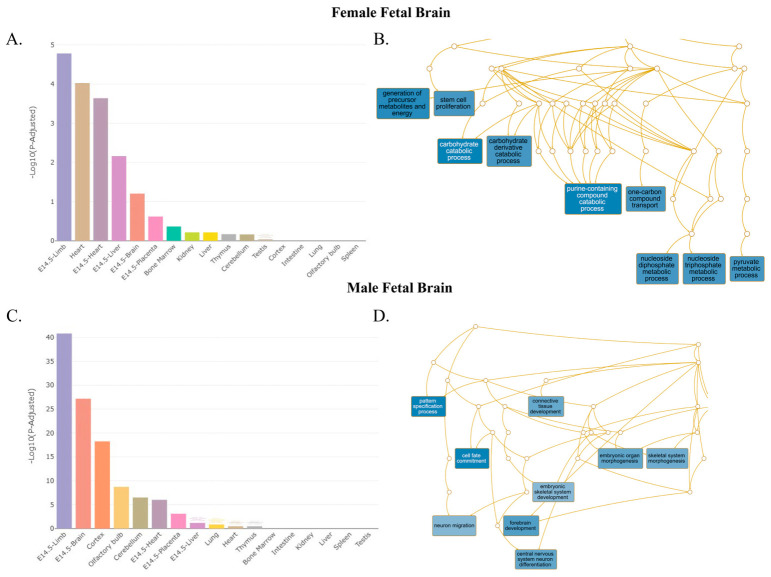
Tissue enrichment and pathways associated with DEGs in female and male fetal brain from probiotic-treated vs. control dams. (**A**) Tissue enrichment for DEGs in female fetal brain for this comparison. (**B**) Pathways associated with DEGs in female fetal brain of probiotic-treated vs. control dams. (**C**) Tissue enrichment for DEGs in male fetal brain of probiotic-treated vs. control dams. (**D**) Pathways associated with DEGs in male fetal brain of probiotic-treated vs. control dams. The color of the box around each pathway indicates statistical significance with light blue indicating a *p* value < 0.05, and dark blue indicating significance at a q value (FDR) < 0.05.

The TissueEnrich program [75] was then used to determine which organs of the mouse have an abundance of transcripts for differentially expressed genes between female and male brain samples. For the female brain, the differentially expressed transcripts are primarily expressed in the limb, heart, liver, brain, and placenta (Figure 6A). For male brain, the differentially expressed transcripts are primarily associated with the limb, brain, cortex, olfactory bulb, heart, and placenta (Figure 6B). Biological processes associated with DEGs in the female brain include ones involved with stem cell differentiation, the carbohydrate catabolic process, the purine-containing compound catabolic process, one carbon compound transport, the nucleoside diphosphate metabolic process, the nucleoside triphosphate metabolic process, and the pyruvate metabolic process (Figure 6C). Those differentially expressed in male brain are associated with forebrain development, neuron migration, central nervous system neuron differentiation, embryonic organ morphogenesis, connective tissue development, and skeletal system morphogenesis (Figure 6D).

### 3.8. Correlation Analyses of Metabolomics and Transcriptomics Data

To determine whether associations existed between amounts of SCFAs within the fetal placenta and brain samples, correlation anlayses were performed. For female placenta, several key transcripts were linked with the amount of SCFAs within this tissue. *Pr4a1* expression negatively correlated with acetic acid in this tissue (Supplementary File S7). *Cecacam9* expression was inversely linked with amounts of isovaleric acid. In contrast, *Sl6a4* expression was positively associated with amounts of this SCFA in the placenta, and there were trends for this transcript to be linked in a positive manner with the other SCFAs examined. In the male placenta, *Pr4a1* and *Pr7a1* inversely correlated with valeric acid. *Hsd11b2* was negatively associated with isovaleric acid (Supplementary File S8). *Dio3* was negatively linked with isovaleric acid and isobutyric acid.

In the female brain, *Actn3*, *Gpx3*, *Pgam2*, *Cox8b*, *Pnpla2*, *Tnni2*, *Acta1*, *Pygm*, *Thrsp*, *Eno3*, and *Fabp4* positively correlated with 2-methylbutanoic acid (Supplementary File S9). *Gh*, *Tnni2, Ucp1*, and *Thrsp* positively associated with isovaleric acid. *Cox8b*, *Pnpla2*, *Acta1*, and *Eno3* were positively linked with butyric acid. *Gpx3* expression positively tracked with isobutyric acid concentrations in this tissue. In male brain samples, amounts of isovaleric acid was positively associated with *Calb2*, *Caly*, *Sncb*, *Scgb3a2*, *Slc6a7*, and *Muc5b* expression (Supplementary File S10). Expression of Igf2bp1 was inversely associated with amounts of 2-methylbutanoic acid and isovaleric acid.

### 3.9. Differentially Expressed Genes Based on Organ and Sex Differences

We next considered how maternal treatment with a probiotic supplement might impact normal sex differences in this organ as well as in the brain. As shown in Figure S7A (page 8 in Supplementary Package), the placenta of probiotic-treated dams had 153 differentially expressed transcripts for females vs. males, whereas control individuals had 98 transcripts that differed for females vs. males. Nine genes overlapped between these two maternal treatment groups (*Hsd3b1*, *Fga*, *Agt*, *Fgb*, *Uty*, *Xist*, *Gm29650*, *Gm4322*, and *Gm28588*, Supplementary File S11). For the fetal brain, 65 transcripts were differentially expressed between males and females in probiotic-treated dams (Supplementary File S12). In contrast 48 genes were differentially expressed in fetal brain samples between males and females from control dams, with 12 transcripts overlapping between the two maternal treatments (*Cryba2*, *Bfsp1*, *Crybb1*, *Crybb3*, *Kdm5d*, *Cryba4*, *Uty*, *Eif2s3y*, *Gfy*, *Rps7-ps3*, *Gm29650*, and *Gm34059*; Figure S7B, page 8 in Supplementary Package; Supplementary File S12). The full list of DEGs for male vs. female placenta from probiotic and control dams are shown in Supplementary Files S13 and S14, respectively. The complete list of DEGs for male vs. female fetal brain from probiotic and control dams are listed in Supplementary Files S15 and S16, respectively.

### 3.10. Target Enrichment and Predicted Diseases/Pathways Based on Organ and Sex Differences

**Figure 7:**
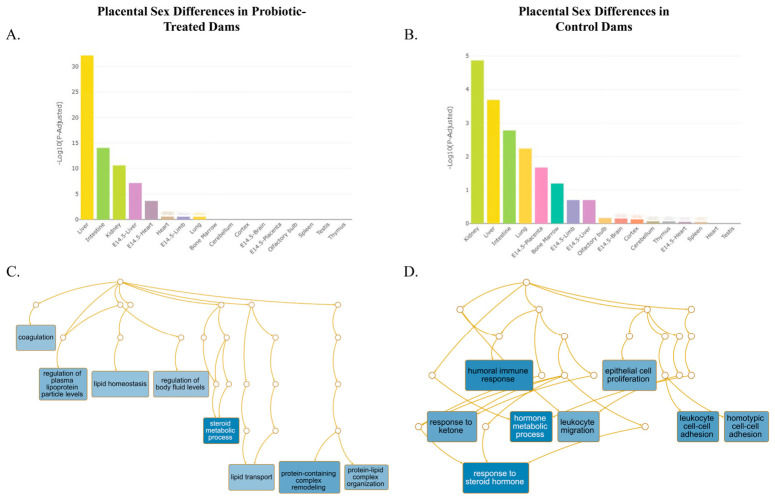
Tissue enrichment and pathways associated with DEGs in placenta based on sex differences in probiotic-treated and control dams. (**A**) Tissue enrichment for DEGs based on sex differences in the placenta of probiotic-treated dams. (**B**) Tissue enrichment for DEGs in placenta based on sex differences in control dams. (**C**) Pathways associated with DEGs in placenta based on sex differences in the placenta of probiotic-treated dams. (**D**) Pathways associated with DEGs in placenta based on sex differences in the placenta of control dams. The color of the box around each pathway indicates statistical significance with light blue indicating a *p* value < 0.05, and dark blue indicating significance at a q value (FDR) < 0.05.

Organs that have enrichment of DEGs in placenta from males vs. females in probiotic-treated dams include the placenta followed by the liver, intestine, kidney, and heart (Figure 7A). Tissue enrichment of DEGs in placenta from males vs. females in control dams reveals that such transcripts are enriched in the kidney, liver, intestine, lung, placenta, bone marrow, and limb (Figure 7B).

Pathways associated with DEGs in placenta from males vs. females in probiotic-treated dams are associated with coagulation, regulation of plasma lipoprotein particle levels, lipid homeostasis, regulation of body fluid levels, the steroid metabolic process, lipid transport, protein-containing complex remodeling, and protein–lipid complex organization (Figure 7C). In contrast, pathways associated with DEGs in placenta from males vs. females of control dams include those associated with humoral immune response, epithelial cell proliferation, response to ketone, the hormone metabolic process, leukocyte migration, leukocyte cell–cell adhesion, and response to steroid hormone (Figure 7D).

**Figure 8:**
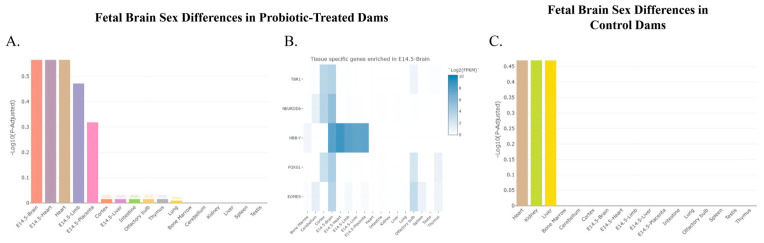
Tissue enrichment and heat map analysis associated with DEGs in fetal brain based on sex differences in probiotic-treated and control dams. (**A**) Tissue enrichment for DEGs based on sex differences in the fetal brain of probiotic-treated dams. (**B**) Heat map analysis of genes enriched in the brain based on DEGs in female brain vs. male brain of probiotic-treated dams. (**C**) Tissue enrichment for DEGs in fetal brain based on sex differences in control dams.

**Figure 9:**
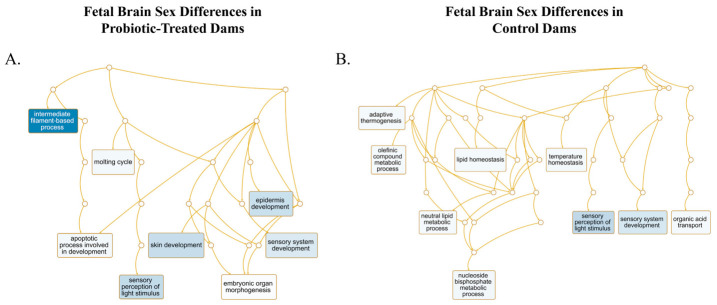
Pathways associated with DEGs in fetal brain based on sex differences in probiotic-treated and control dams. (**A**) Pathways associated with DEGs based on sex differences in the fetal brain of probiotic-treated dams. (**B**) Pathways associated with DEGs in fetal brain based on sex differences in control dams. The color of the box around each pathway indicates statistical significance with light blue indicating a *p* value < 0.05, and dark blue indicating significance at a q value (FDR) < 0.05.

Transcripts that are differentially expressed in fetal brain of males vs. females from probiotic-treated dams are enriched in the embryonic brain, heart, limb, and placenta (Figure 8A). Transcripts enriched in the embryonic brain include *Tbr1*, *Neurod6*, *Hbb-g*, *Foxg1*, and *Eomes* (Figure 8B). For fetal brain comparison of males vs. females in control dams, DEGs are associated with the heart, kidney, and liver but surprisingly not any neural tissues (Figure 8C). Pathways with DEGs in fetal brain from males vs. females from probiotic-treated dams include the intermediate filament-based process, apoptotic process involved in development, sensory system development, sensory perception of light, and embryonic organ development (Figure 9A). The DEGs in fetal brain of males vs. females from control dams are linked to adaptive thermogenesis, lipid homeostasis, the neutral lipid metabolic process, the nucleoside biphosphate metabolic process, temperature homeostasis, sensory perception of light stimulus, sensory system development, and organic acid transport (Figure 9B).

## 4. Discussion

The primary goal of this study was to determine whether maternal probiotic supplementation might impact the placenta–brain axis. As part of these studies, bacterial SCFAs were examined in the stool samples of pregnant mice who received this supplementation and compared to concentrations in control pregnant mice. The same SCFAs were then examined in the placenta and fetal brain from conceptuses derived from both maternal groups. Lastly, transcriptomic profiles in the placenta and fetal brain were examined to determine if maternal probiotic supplementation might induce molecular alterations in these organs that could impact conceptus development. In relation to the first aim, no differences in SCFAs were detected between the two groups of pregnant mice. Female mice were provided the probiotic supplement two weeks prior to breeding until conceptuses were collected. Had the females been provided this supplement for a longer duration, it is possible that changes in SCFAs would be observed in the stool and possibly the plasma. The previous study that we based the current work on provided the same probiotic treatment from prior to pregnancy through lactation and reported that this supplement increased the amount of gut (fecal) acetate and butyrate levels and plasma lactate levels [27]. Thus, the bacteria in this supplement can synthesize these SCFAs. We used the exact same number of replicates as in this previous work. One other possible explanation for the differences between the current and previous study is that we provided this supplement to C57Bl6J mice, whereas CD1 mice were used in the prior experiments [27].

Men placed on a synbiotic treatment, which included six species of probiotic microorganisms, for twelve weeks had increased concentrations of acetate, butyrate, propionate, and valerate [76]. It is possible that while this treatment did not affect SCFA concentrations in the stool of pregnant mice, other beneficial bacterial metabolites, such as polyamines (PAs, which include putrescine, spermidine and spermine) and vitamins B9 and B12 might have been altered. Such metabolites will be the focus of future research. In a recent review we highlight how each of the other metabolites might also impact placental function by impacting DNA stability, placental growth, and angiogenesis [26].

Examination of SCFAs within the placenta and fetal brain also did not reveal significant changes with the one exception that valeric acid was reduced in the fetal brain of conceptuses derived from probiotic-treated dams (Figure 1). The underlying mechanism for this reduction and significance of this finding is uncertain. Other studies examining direct probiotic supplementation have shown an increase in valeric acid that is associated with beneficial behavioral effects [71,72,77]. It is possible that maternal probiotic supplementation might lead to long term changes in neural SCFA concentrations that are linked with positive behavioral traits. A study with maternal probiotic treatment to obese mice increased gut butyrate and brain lactate in juvenile and adult offspring [27]. This supplementation also reduced anxiety-like behaviors in offspring, which correlated with elevations in brain lactate concentrations. Thus, further work is needed to characterize how maternal probiotic supplementation influences SCFAs and other bacterial metabolites in the placenta throughout pregnancy and in the brain of offspring throughout their lifespan.

Even though no differences were noted in maternal fecal boli, placenta, and fetal brain samples for the SCFAs, current results in terms of distribution of the SCFAs mirror those reported previously [78,79] with the primary SCFA being acetic acid followed by propionic acid. In pregnant women, fiber supplementation altered gut bacterial species but did not impact circulating SCFA [80]. Further, treatment of placental explants with propionate had no effect on key placental pathways [80], further suggesting that the effect of maternal gut microbiota changes on the placenta might operate through other metabolites or mechanisms.

Substantial other work suggests that SCFAs are integral to normal placental responses. Placenta from conceptuses derived from antibiotic treatment of pregnant mice are reduced in size and have reduced vascularization that likely results in decreased transport of nutrients, oxygen, and immune molecules from mother to conceptus [81]. Correspondingly, these fetuses show reductions in SCFAs [81]. SCFA supplementation in malnourished pregnant mice mitigated the placental growth restriction and vascular deficiency that would otherwise exist [81]. Maternal SCFA treatment decreased chemotaxis of placental neutrophils [62]. Butyrate concentrations are 3–4x elevated in cows with normal expulsion of the extraembryonic membranes [82]. In healthy pregnant women and those with gestational diabetes, circulating acetate, propionate, and butyrate correlate with placental function and fetal development at birth, and such effects might be modulated through G-protein-coupled receptors (GPCRs) or histone deacetylases (HDACs) [62], suggesting that supplementation of these bacterial metabolites might be beneficial to the placenta and conceptus. SCFAs alter placental expression of tryptophan 5-hydroxylase 1, an enzyme essential for serotonin synthesis [58]. The mouse and human placenta appear to produce serotonin that regulates early forebrain development [20,22,59,60,61]. SCFAs stimulate proliferation of human neural progenitor cells [62].

While minimal effects of maternal probiotic supplementation were noted on SCFAs in the placenta and fetal brain, conceptuses derived from these dams had notable transcriptomic alterations in the placenta and fetal brain. Sex-dependent differences in the placenta and fetal brain were observed in response to maternal probiotic treatment. In the placenta, this maternal supplementation led to a greater number of gene expression changes in females. Conversely, the fetal brain of males showed the most dramatic changes in response to this maternal treatment.

DEGs in the placenta of females and males from probiotic-treated dams compared to control dams were primarily enriched in the placenta and included several *Prl* forms, including *Prl7a1* and *Prl6a1*. Both prolactin forms were decreased in female and male placenta from maternal probiotic-treated dams vs. conceptuses from control dams. In the rodent placenta, prolactins are linked with maternal recognition of pregnancy and appear to be essential for normal placental development [83,84]. Alteration of these gene forms and concomitant decrease in protein expression might thus lead to maternal and placental dysfunction.

Male placenta from probiotic-treated dams also had reduced expression of *Hsd11b2*. The protein encoded by this gene is abundant in human syncytiotrophoblast cells of placental villi and labyrinthine TB of the mouse placenta [85,86,87,88,89]. 11B-HSD2 catalyzes the oxidation of maternally derived cortisol to cortisone (an inactive form) that is then transferred to the fetus [85,86,87,88]. Thus, 11B-HSD2 protects the fetus from elevated maternal glucocorticoids, which can be brought upon by stress and alterations in this enzyme are linked with several human gestational disorders, including intrauterine growth restriction, impaired fetal growth, and anxiogenic behavior [85,86,87,88,90].

One gene that was increased in the female placenta derived from probiotic-treated dams was *Slc6a4*, which encodes for the serotonin transporter (SERT). Kliman et al. [91] have suggested that *Slc6a4*/SERT is the primary mediator of 5-HT transport from the maternal side to the placenta. 5-HT can impact placenta growth and function. During early fetal development, the placenta provides the initial source of 5-HT to the emerging forebrain region [20,92,93]. Thus, factors that might impact the ability of the placenta to accrue maternal 5-HT can also impact brain development.

In both male and female placenta, likely pathways affected by maternal probiotic supplementation include cell killing and regulation of body fluid levels. Such pathways might influence general morphology, TB cell numbers and differentiation, and nutrient acquisition. The net effects of such alterations might also impact conceptus development and the ability of the placenta to provide vital nutrients to the brain and other fetal organs.

Maternal probiotic supplementation results in dramatic transcriptomic changes in the fetal brain. In contrast to the placenta, the brain of male conceptuses appears to be more sensitive to this maternal treatment. It is not clear though whether such changes are due to direct effects of this maternal treatment or originate because of probiotic-induced placental disruptions, a point discussed further below. The net effect though is that the cumulative transcriptomic alterations in the brain of males are associated with pathways guiding forebrain development and neuron migration and differentiation. Differentially expressed genes in the brain of female conceptuses, however, are associated with metabolic pathways, including those associated with carbohydrates, nucleosides, and purines.

A recent meta-analysis concluded that maternal microbiota changes, which could be brought upon by probiotic supplementation, provide compelling evidence that they can impact fetal brain development through immune, metabolic, epigenetic, and hormonal pathways [94]. Besides SCFAs, maternal microbiome alterations can influence fetal brain development through bile acids, tryptophan-based compounds, maturation of microglial cells (resident macrophages in the brain), compromising the blood–brain barrier, and neurogenesis. Prenatal exposure to antibiotics has been linked with higher rates of autism spectrum disorders (ASDs) and attention-deficit/hyperactivity disorder (ADHD) [94].

How maternal probiotic treatment leads to transcriptomic changes in the male and female placenta and fetal brain samples remains uncertain. One likely possibility is that bacterial metabolites might modulate such changes. While we did not see significant changes in the SCFAs measured, the correlation analyses reveal clear linkages between each SCFA and gene expression changes for the different comparisons. Notably, in the female placenta the amount of isovaleric acid positively correlated with the expression of *Slc6a4*, the serotonin transporter (detailed above), and the remaining SCFAs showed trends for positive correlation with this transcript. In the male placenta, *Pr4a1* and *Pr7a1* inversely correlated with valeric acid. *Hsd11b2* was negatively associated with isovaleric acid. Dio3 was negatively linked with isovaleric acid and isobutyric acid. It is possible that other bacterial metabolites might drive such gene expression changes. Prior studies with GF pregnant females provided a probiotic and others that did not receive this supplement and findings in pregnant women treated with a probiotic supplement provide support for the notion that changes in the maternal gut microbiome and possibly in bacterial metabolites influence gene expression patterns in the placenta and other fetal tissues/samples. Treating GF pregnant mice with *Bifidobacterium breve* UCC2003 led to changes in the placental architecture and accompanying alterations in nutrient transport and fetal growth [31]. The placentas of conceptuses from these dams also show metabolite changes in the placental junctional zone, upregulated expression of nutrient carriers, in particular *Slc7a8* and *Slc16a4*, and increased amounts of prolactins and pregnancy-specific glycoproteins relative to control GF dams. Angiogenesis and associated factors showed disruptions in the junctional and labyrinth zones for placentas derived from GF dams not treated with any probiotic [32]. Prolactin genes are suppressed in the placenta of conceptuses carried by pregnant untreated GF dams [33]. The term placentas of conceptuses derived from pregnant women provided a probiotic supplement containing *Bifidobacterium lactis* alone or in combination with *Lactobacillus rhamnosus GG (LGG)* demonstrate altered expression of toll-like receptor (TLR)-related genes in the placenta and in the fetal gut (meconium) [34].

Previous work in female mice that we based our study design on has shown that exposure to this probiotic increases the amount of fecal *Lactobacillus* spp. and *Bifidobacterium* spp. relative to control females [27]. Thus, we assume the same should be the case for our current work. In this aspect, there is considerable overlap in gastrointestinal bacteria across species spanning mice to humans [95,96]. Admittedly, it would have been helpful to confirm this to be the case, especially as we chose to examine C57B6J mice as opposed to the CD1 mice that were used in the original study. Future work will include running comprehensive gut microbiota analyses. In the current studies, we focused on examining SCFAs in maternal fecal samples where we did not detect significant differences. Potential differences in SCFAs between maternal probiotic-supplemented compared to control dams might have been detected in maternal plasma or serum. Thus, future work will include measuring the same SCFAs in maternal circulation. It is possible that differences in SCFAs will be detected when female mice are provided the probiotic supplement for a longer time prior to breeding. Another limitation of the current study is that it is not clear whether the findings in the brain are due to direct effects of maternal probiotic supplementation or through indirect effects via the placenta–brain axis. It is seemingly impossible at the current time to dissect whether the net changes are attributed to one or both pathways as any maternal treatment will simultaneously impact both organs. If it can be discerned whether select bacterial metabolites are correlated with changes in fetal brain expression, it may be possible to use ex vivo or in vitro approaches to examine the effects of such metabolites in fetal neuron cultures. Such changes could then be compared to those identified in the whole animal model to parse out direct vs. indirect effects of maternal probiotic on the fetal brain. Conversely, fetal brain cells could seemingly be co-cultured with primary TB cells or treated with their products, such as exosomes, following placental exposure to maternal probiotic supplementation. Such an approach would permit examination whether maternal probiotic treatment results in indirect effects through the placenta–brain axis. This term has gained currency as it is increasingly becoming clear that factors, including serotonin, from the placenta can influence fetal brain development [22]. It also implies that the close connection between these two organs that changes in the placenta can lead to immediate and potentially long-term impact on neurobehavioral patterns.

The placenta and fetal brain demonstrate clearly sexually dimorphic differences [22,44,97,98,99,100,101,102,103,104,105]. Maternal probiotic supplementation also impacts such sex differences in gene expression in the placenta and fetal brain. In control placenta, sex differences in gene expression were associated primarily with pathways regulating immune function, such as humoral immune responses, leukocyte migration, and leukocyte cell–cell adhesion. In placenta from maternal probiotic-treated dams, differentially expressed genes based on sex were linked with metabolic and steroid processes, including lipid transport, lipid homeostasis, and lipid-protein complexes. Sex differences in control fetal brain correlated with pathways regulating lipid metabolism and transport, whereas those from dams treated with maternal probiotic supplementation associated primarily with the intermediate filament pathway. The long-term effects of such alterations on existing sex differences in both organs remains to be determined.

Future studies include examining how maternal probiotic supplementation might influence long-term offspring behavioral traits. Other studies indicate this prenatal treatment might lead to beneficial behavioral effects, including social and emotional behaviors [27,106,107]. We are also currently examining how ablation of the maternal gut microbiome in GF mice influences the placenta–brain axis. Results of these ongoing studies will provide further support on the role of the maternal gut microbiome in guiding development and function of both organs.

From a clinical perspective, current studies suggest the need for further human studies to examine how maternal probiotic supplementation might impact the placenta and later offspring responses, including neurobehavioral traits. A potential non-invasive mechanism to explore effects on the placenta would be to screen placental-derived extracellular vesicles circulating in the maternal serum. This approach has been widely used to examine how gestational disorders, such as pre-eclampsia and gestational diabetes impact placenta function [108]. As the mouse and human placenta share commonalities, the current findings suggest that maternal probiotic supplementation might influence placental morphology and pathophysiological responses. Consequently, clinicians should consider discussing with pregnant women the potential beneficial and adverse effects such supplements might have on them and their unborn offspring.

In conclusion, the current work suggests that short-term probiotic supplementation of periconceptional/gestational dams does not influence bacterial SCFAs in maternal fecal samples or the placenta and fetal brain of conceptuses derived from such females. It remains to be determined whether such treatment alters other bacterial metabolites. While this treatment did not affect bacterial SCFAs, placenta and fetal brain changes were evident in male and female conceptuses carried by dams receiving probiotics relative to controls. In both organs, transcriptomic alterations were sex-dependent. For the placenta, females were more sensitive to maternal probiotic supplementation, whereas the opposite was the case for the fetal brain. *Slc6a4* showed increased expression in female placenta from probiotic-treated dams, which could enhance uptake of maternal 5-HT. Male placenta from the same group had a dramatic reduction in *Hsd11b2* that may render them more vulnerable to maternal stress. Several key placental *Prl* forms were reduced in both male and female placenta derived from maternal dams treated with a probiotic supplement. In the fetal brain, maternal probiotic supplementation was associated with genes linked to forebrain development and neuronal differentiation, suggesting this treatment might impact immediate and life-long neurobehavioral responses, although this was not assessed in the current work. The degree to which maternal probiotic supplementation operates through the placenta–brain axis remains to be determined. Current studies suggest caution on usage of probiotic supplements by pregnant mothers. While such treatment might lead to beneficial effects on the mothers, they can also, for better or worse, influence the placenta and fetal brain of their unborn children.

## Figures and Tables

**Table 1:** Top five most up- and downregulated transcripts of biological relevance in female placenta from probiotic-treated dams vs. control dams with a p-adj (q-value) ≤ 0.05.

Gene Symbol	Fold Change (Probiotic-Treated vs. Control Dams)	p-adj (q Value)	General Function of the Encoded Protein
**Top Downregulated Transcripts of Biological Relevance**			
*Aqp5*	0.002992	0.00653	Modulate water transport across the plasma membrane along its osmotic gradient.
*Guca2b*	0.006076	0.00653	Binding of this peptide to a receptor leads to an increase in cyclic GMP, which regulates salt and water homeostasis.
*Akr1b7*	0.009753	0.00653	Catalyzes the NADPH-dependent reduction of several compounds to corresponding alcohols. Endogenous metabolites it can act upon include aromatic and aliphatic aldehydes, ketones, monosaccharides, bile acids and xenobiotics substrates.
*Pck1*	0.022999	0.00653	Catalyzes the formation of phosphoenolpyruvate from oxaloacetate via phosphoryl donor GTP, releasing carbon dioxide. This protein is important for the regulation of gluconeogenesis.
*Pigr*	0.023108	0.00653	Immunoglobin receptor found at the basolateral membrane of epithelial cells that binds polymeric immunoglobulin to be secreted at the apical membrane.
**Top Upregulated Transcripts of Biological Relevance**			
*Psg20*	8.036069	0.00653	Member of the immunoglobulin superfamily. Produced by placental trophoblasts to possibly regulate maternal immune and vascular functions via receptor binding and modulation of cytokine and chemokine activity.
*Slc6a4*	2.799424	0.00653	Enables serotonin–sodium–chloride symporter activity. Regulation of this symporter modulates serotonin cellular uptake.
*Slc30a3*	2.681109	0.016964	Enables zinc ion importer activity in the synaptic vesicle. Acts upstream of positive regulation of transport.
*Wnt9b*	2.47433356	0.0492664	Encodes for a secreted signaling protein part of the Wnt/ -catenin signaling pathway that guides embryonic development, placenta formation, trophoblast differentiation, and implantation.
*Prl5a1*	2.316971021	0.00653032	Encodes for a protein that regulates uterine natural killer cells and adaptation to uterine stress and may be part of a larger family of molecules that regulates maternal recognition of pregnancy in rodents.

**Table 2:** Top five most up- and downregulated transcripts of biological relevance in male placenta from probiotic-treated dams vs. control dams with a p-adj (q-value) ≤ 0.05.

Gene Symbol	Fold Change (Probiotic-Treated vs. Control Dams)	p-adj (q Value)	General Function of the Encoded Protein
**Top Downregulated Transcripts of Biological Relevance**			
*Dio3*	0.049858	0.018443	Member of the iodothyronine deiodinase family. Regulates thyroid hormone concentrations by catalyzing the inactivation of thyroid hormone via deiodination of the prohormone thyroxine (T4) and the bioactive hormone 3,3′,5-triiodothyronine (T3) to inactive metabolites, 3,3′,5′-triiodothyronine (RT3) and 3,3′-diiodothyronine (T2), respectively.
*Hsd11b2*	0.08708	0.018443	Catalyzes the conversion of cortisol to inactive cortisone form. Protects placental cells from growth-inhibiting and pro-apoptotic effects of cortisol during embryonic development.
*Slc38a8*	0.143289	0.018443	Sodium-dependent amino-acid/proton antiporter with eleven transmembrane domains, an extracellular N-terminus and an intracellular C-terminal tail.
*Gzmd*	0.193826804	0.018443	Encodes for a serine protease involved in immune responses. In the placenta, it may respond to nutrient restrictions or inflammation.
*Prf1*	0.201986391	0.018443	The protein encoded by this gene may regulate maternal immune tolerance to the conceptus. Dysfunctions of this protein are associated with placental inflammation and abnormal natural killer cell activity.
**Top Upregulated Transcripts of Biological Relevance**			
*Eln*	3.058258	0.018443	Elastin is one of the two components of elastic fibers. Elastic fibers comprise part of the extracellular matrix and confer elasticity to organs and tissues.
*Kcnh1*	2.865943	0.018443	Member of the potassium channel, voltage-gated, subfamily H. This protein is a pore-forming (alpha) subunit of a voltage-gated non-inactivating delayed rectifier potassium channel. It is activated by onset of myoblast differentiation.
*Col6a2*	2.690305	0.018443	One of the three alpha chains of type VI collagen, a beaded filament found in most connective tissues. This protein contains several domains shown to bind extracellular matrix proteins.
*Col6a1*	2.49021447	0.018443	Reduced expression of this gene that encodes for collagen type VI alpha 1 is associated with impaired placental development, including decreased trophoblast migration and angiogenesis within the placenta.
*Hspa1a*	2.400159031	0.018443	This gene encodes for heat shock protein 70 (Hsp70) that stabilizes the placental function under stressful conditions.

**Table 3:** Top five most up- and downregulated transcripts of biological relevance in female brain from probiotic-treated dams vs. control dams with a p-adj (q-value) ≤ 0.05.

Gene Symbol	Fold Change (Probiotic-Treated vs. Control Dams)	p-adj (q Value)	General Function of the Encoded Protein
**Top Downregulated Transcripts of Biological Relevance**			
*Ahcyl*	0.017957	0.024998	Possibly involved in the conversion of S-adenosyl-L-homocysteine to L-homocysteine and adenosine.
*Hsd3b6*	0.038789	0.024998	May enable 3-beta-hydroxy-Delta5-steroid dehydrogenase (NAD+) activity and steroid Delta-isomerase activity.
*Erv3*	0.054949	0.024998	A conserved protein with a predicted signal peptide and similarity to the Env polyprotein. This protein is overexpressed in colorectal and other cancers.
*Tal2*	0.166828	0.024998	May enable RNA polymerase II-specific and RNA polymerase II cis-regulatory region sequence-specific DNA-binding activity.
*Slc4a1*	0.168254	0.024998	Chloride/bicarbonate exchanger in the erythrocyte plasma membrane involved in carbon dioxide transport from tissues to lungs. This protein is associated with erythrocyte membrane protein glycophorin A and promotes the correct folding and translocation of the exchanger.
**Top Upregulated Transcripts of Biological Relevance**			
*Pomc*	7.566573	0.024998	Preproprotein that undergoes extensive, tissue-specific, post-translational processing via prohormone convertases. Depending on tissue type and the available convertases, processing can yield up to ten active peptides involved in diverse cellular functions, including adrenocorticotropic hormone (ACTH), β-endorphin, melanocyte-stimulating hormone (MSH), and β-lipotropin (LPH).
*Thrsp*	7.490852943	0.024998	This gene encodes for the thyroid hormone-responsive SPOT 14 protein that regulates lipid metabolism. Overexpression in the brain may lead to altered dopamine signaling, memory deficits, attention deficit disorders, and cognitive changes.
*Gh*	6.395069331	0.024998	Growth hormone (GH) is essential for normal brain development, neurogenesis, synaptic plasticity, memory, cognitive function, and neuroprotection.
*Cox8b*	5.015383429	0.024998	Encodes for cytochrome c oxidase subunit 8B, which is a nuclear-encoded subunit of the mitochondrial electron transport chain complex IV, essential for regulation of cellular respiration and energy production.
*Cdh26*	4.438093309	0.024998	Encodes for a calcium-dependent cell-adhesion protein that is crucial for cell–cell junction organization and structure.

**Table 4:** Top five most up- and downregulated transcripts of biological relevance in male brain from probiotic-treated dams vs. control dams with a p-adj (q-value) ≤ 0.05.

Gene Symbol	Fold Change (Probiotic-Treated vs. Control Dams)	p-adj (q Value)	General Function of the Encoded Protein
**Top Downregulated Transcripts of Biological Relevance**			
*Hsd3b6*	0.032856	0.00812	Modulates 3-beta-hydroxy-Delta5-steroid dehydrogenase (NAD+) activity and steroid Delta-isomerase activity. Likely involved in C21-steroid hormone biosynthesis, androgen biosynthetic process, and negative regulation of iron ion transport.
*Aqp8*	0.043402	0.00812	Modulates water transport across the plasma membrane along its osmotic gradient.
*Tal2*	0.141183	0.00812	May enable DNA-binding transcription factor activity and RNA polymerase II-specific and cis-regulatory region DNA-binding activity.
*Ahcyl*	0.162911	0.018875	Involved in the conversion of S-adenosyl-L-homocysteine to L-homocysteine and adenosine.
*Hmga2*	0.181807	0.00812	Functions as an architectural factor and essential component of the enhancesome. Contains structural DNA-binding domains and may function as a transcriptional regulating factor.
**Top Upregulated Transcripts of Biological Relevance**			
*Fezf2*	5.17765917	0.00812	Encodes for Fez family zinc finger 2 that is a critical transcription factor gene for brain development, including formation of subcortical projection neurons and corticospinal motor neurons in the cerebral cortex.
*Neurod6*	4.85412076	0.00812	Neurogenic Differentiation 6 is a basic helix–loop–helix transcription factor gene vital for brain development, neuronal differentiation, and survival, especially in the neocortex, hippocampus, and midbrain dopaminergic neurons
*Foxg1*	4.793967073	0.00812	Encodes for a transcription factor that is critical for early forebrain development, including neural cell proliferation and brain patterning.
*Tbr1*	4.708181705	0.00812	The T-box brain 1 gene encodes for a transcription factor important for brain development, especially neurogenesis, neuronal migration, and laminar structure, in the cerebral cortex, amygdala, and olfactory bulb. Deficiencies of this gene are linked with autism spectrum disorders and intellectual disability.
*Neurod2*	4.196597211	0.00812	Encodes for a calcium-dependent transcription factor critical in regulating structural and functional maturation of the hippocampal mossy fiber (MF) synapse.

## Data Availability

The RNA sequencing data presented in this study are openly available in the Gene Expression Omnibus under accession: GSE309308 (https://www.ncbi.nlm.nih.gov/geo/query/acc.cgi?acc=GSE309308, accessed on 16 March 2026).

## Supplementary Materials

The following supporting information can be downloaded at: https://www.mdpi.com/article/10.3390/microorganisms14061175/s1, **Figure S1:** Diagram summarizing experimental studies; **Figure S2:** Maternal body weight prior to and during pregnancy and water consumption for dams placed on vehicle control and those provided probiotic supplement; **Figure S3:** 2D PCA plot of SCFA in maternal fecal samples prior to and after probiotic treatment and in vehicle controls; **Figure S4:** 2D PCA plot of SCFA in placenta and fetal brain samples from probiotic and control dams; **Figure S5:** 2D PCA plot of RNAseq results from placenta and fetal brain samples from probiotic and control dams; **Figure S6:** Volcano plots showing differentially expressed genes (DEG) for female and male placenta and fetal brain from maternal probiotic treated vs. counterpart controls; **Figure S7:** Venn diagram comparisons of sex differences in maternal probiotic-treated and control dams for placenta and fetal brain samples; **Table S1:** Details on probiotic, Bio-Kult^®^ Advanced, ADM Protexin Ltd.; 14 bacterial strains plus supplements, used in current study; **Table S2:** The MRM parameters for each short chained fatty acid (SCFA) examined; **Table S3:** Number of raw reads, mapped reads, and % mapped reads for RNA seq results from placenta and fetal brain from maternal probiotic and control treated mice; **Table S4:** Results for all metabolites in fecal boli samples from control and probiotic-treated mouse dams. Results are reported in ng/mg of tissue; **Table S5:** Results for all metabolites in placenta and fetal brain samples from control and probiotic-treated mouse dams. Results are reported in ng/mg of tissue; **Supplementary File S1.** Venn diagram comparison of DEGs in female placental comparison vs. male placental comparison; **Supplementary File S2:** Complete list of DEGs in female placenta from conceptuses derived from probiotic treated dams vs. female placenta from conceptuses derived from control dams; **Supplementary File S3:** Complete list of DEGs in male placenta from conceptuses derived from probiotic treated dams vs. male placenta from derived conceptuses derived from control dams; **Supplementary File S4:** Venn diagram comparison of DEGs in female fetal brain comparison vs. male fetal brain comparison; **Supplementary File S5:** Complete list of DEGs in female fetal brain from conceptuses derived from probiotic treated dams vs. female fetal brain from conceptuses derived from control dams; **Supplementary File S6:** Complete list of DEGs in male fetal brain from conceptuses derived from probiotic treated dams vs. male fetal brain from conceptuses derived from control dams; **Supplementary File S7:** Correlation analysis of differentially expressed transcripts in female placenta derived conceptuses from probiotic treated dams vs. female placenta from conceptuses derived from control dams and mount of SCFAs within this tissue; **Supplementary File S8:** Correlation analysis of differentially expressed transcripts in male placenta derived conceptuses from probiotic treated dams vs. male placenta from conceptuses derived from control dams and mount of SCFAs within this tissue; **Supplementary File S9:** Correlation analysis of differentially expressed transcripts in female fetal brain derived conceptuses from probiotic treated dams vs. female fetal brain from conceptuses derived from control dams and mount of SCFAs within this tissue; **Supplementary File S10:** Correlation analysis of differentially expressed transcripts in male fetal brain derived conceptuses from probiotic treated dams vs. male fetal brain from conceptuses derived from control dams and mount of SCFAs within this tissue; **Supplementary File S11.** Venn diagram comparison of sex differences in the placenta of probiotic-treated dams vs sex differences in the placenta from control dams. 144 transcripts differed based on sex in probiotic treated dams, whereas control individuals have 98 transcripts that differed based on sex. Unique genes that differed based on sex in probiotic treated dams are shown in Column A, and those that were unique for control dams are shown in Column C. Nine genes overlapped between these two maternal treatment groups (*Hsd3b1*, *Fga*, *Agt*, *Fgb*, *Uty*, *Xist*, *Gm29650*, *Gm4322*, and *Gm28588*), and are shown in Column B; **Supplementary File S12:** Ven diagram comparison of sex differences in the fetal brain of probiotic treated dams vs. sex differences in the fetal brain from control dams. Five transcripts were differentially expressed between males and females in probiotic-treated dams. In contrast 48 genes were differentially expressed in fetal brain samples between males and females from control dams. Unique genes that differed based on sex in probiotic treated dams are shown in Column A, and those that were unique for control dams are shown in Column C. Twelve transcripts overlapping between the two maternal treatments (*Cryba2*, *Bfsp1*, *Crybb1*, *Crybb3*, *Kdm5d*, *Cryba4*, *Uty*, *Eif2s3y*, *Gfy*, *Rps7-ps3*, *Gm29650*, and *Gm34059*), and these are shown in Column B; **Supplementary File S13.** Complete list of DEGs in female placenta from conceptuses derived from probiotic treated dams vs. male placenta from conceptuses derived from probiotic treated dams; **Supplementary File S14:** Complete list of DEGs in female placenta from conceptuses derived from control dams vs. male placenta from conceptuses derived from control dams; **Supplementary File S15:** Complete list of DEGs in female fetal brain from conceptuses derived from probiotic treated dams vs. male fetal brain from conceptuses derived from probiotic treated dams; **Supplementary File S16:** Complete list of DEGs in female fetal brain from conceptuses derived from control dams vs. males fetal brain from conceptuses derived from control dams.
