# Supplementary material for: Susceptibility of the Placenta and Fetal Brain to Maternal Probiotic Supplementation
Source: Microorganisms. 2026 May 22;14(6):1175. doi: 10.3390/microorganisms14061175 (PMC13303452; doi:10.3390/microorganisms14061175)
Supplement: Supplementary file 1 [file microorganisms-14-01175-s001.zip › Supplementary Package_Figures S1–S7 and Tables S1–S5_5_8_26.pdf]

Supplementary Material

Susceptibility of the Placenta and Fetal Brain to Maternal Probiotic Supplementation

Rosalind T.B. Herrington[1,2], Zhen Lyu[3,4], David T. Ellenberger[1]
Nathan J. Bivens[5], Zhentian Lei[6,7], Tanhaul Islam[6,7], Lloyd W. Sumner[6,7],
R. Michael Roberts[7,8], Trupti Joshi[3,4,9,10], and Cheryl S. Rosenfeld[1,10,11,12]

Departments of:
[1]Pathobiology and Integrative Biomedical Sciences. University of Missouri, Columbia, Missouri, 65211, USA
[2]Biological Sciences, University of Missouri, Columbia, Missouri, 65211, USA
[3] Department of Biomedical Sciences, Joan C. Edwards School of Medicine, Marshall University, Huntington, West Virginia, USA
[4] Christopher S. Bond Life Science Center, University of Missouri, Columbia, MO, 65211, USA
[5]Genomics Technology Core Facility, University of Missouri, Columbia, Missouri, 65211, USA
[6]University of Missouri Metabolomics Center, University of Missouri, Columbia, MO 65211.
[7]Biochemistry, University of Missouri, Columbia, MO 65211.
[8]Animal Sciences, University of Missouri, Columbia, Missouri, 65211, USA
[9]Department of Biomedical Informatics, Biostatistics, and Medical Epidemiology, School of Medicine, University of Missouri-Columbia, MO, 65201, USA
[10]MU Institute for Data Science and Informatics, University of Missouri, Columbia, Missouri, 65211, USA
[11]Department of Genetics Area Program, University of Missouri, Columbia, Missouri, 65211, USA
[12]Department of Thompson Center for Autism and Neurobehavioral Disorders, University of Missouri, Columbia, Missouri, 65211. USA

Short Title: Probiotic Effects on the Placenta-Brain Axis

Keywords: Trophoblast, Pregnancy, Gut Microbiome, Bacterial Metabolites, Transcriptomics, Metabolomics, Short-Chained Fatty Acids, SCFA

Correspondence: Joshitr@marshall.edu or rosenfeldc@missouri.edu

Supplementary Figures

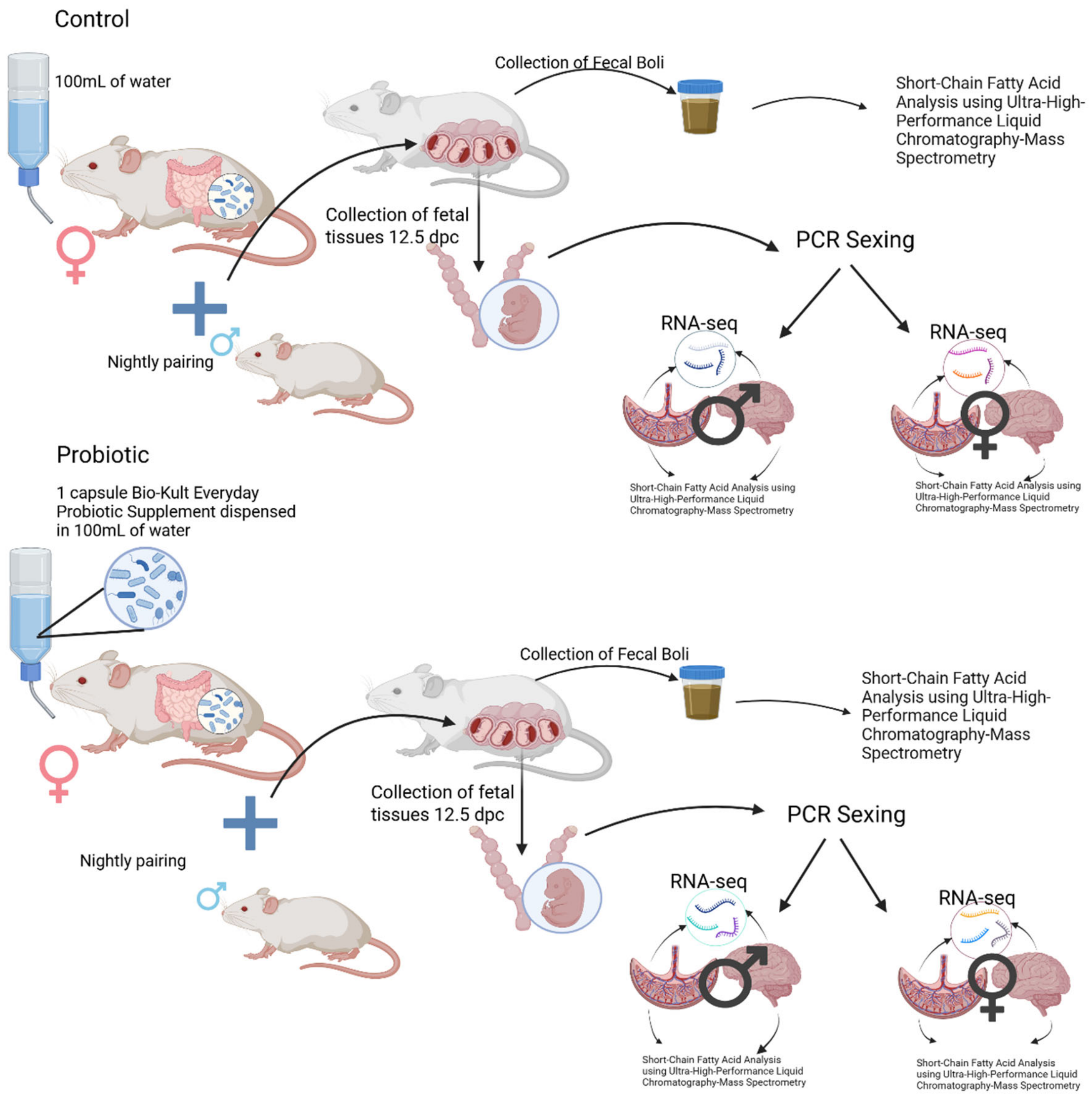

Figure S1. Diagram summarizing experimental studies. As the diagram shows, there were two groups of female mice: those who were provided water only (control) and those where the probiotic supplement was added to the water. These treatments were initiated two weeks prior to breeding to male mice. Day of the vaginal plug was considered 0.5 day post-coitus (dpc). Female had there stool collected and were euthanized at 12.5 dpc. The position of the conceptuses in the uterine horn was documented. Portion of the fetal tissue was collected for PCR sexing based on presence or absence of *Sry*. The placenta and fetal brain were divided between tissue that was to have RNA isolated and submitted for RNAseq analyses and the other half for metabolomics analyses of the SCFA. The same procedures were followed for both groups of pregnant female mice.

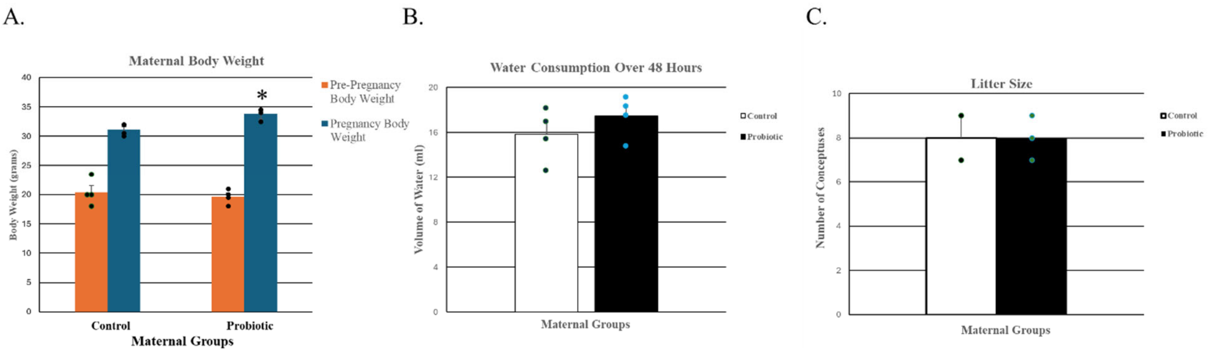

Figure S2. Maternal body weight prior to and during pregnancy and water consumption for dams placed on vehicle control and those provided probiotic supplement. A) Female mice placed on vehicle control and probiotic diet showed no weight differences prior to being placed on these treatments. However, pregnant dams on probiotic supplement weighed more than control dams at time of euthanasia/conceptus collection. B) Pregnant mice on probiotic supplement drank the same amount over 48 hours as control dams. Probiotic supplement/vehicle control treated water was changed every 48 hours. *p = 0.008. C) Litter weights did not differ for controls and dams on the probiotic treatment. N = 4 female mice/group. Individual data points are shown for each group.

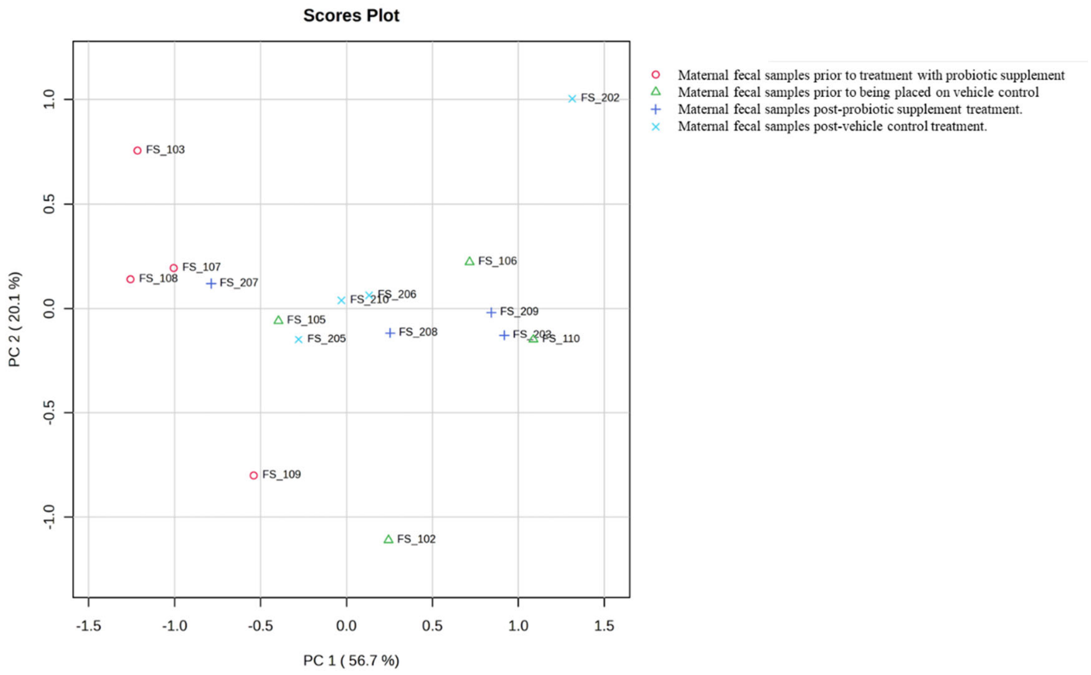

Figure S3. 2D PCA plot of SCFA in maternal fecal samples prior to and after probiotic treatment and in vehicle controls. N= 4 replicates/group.

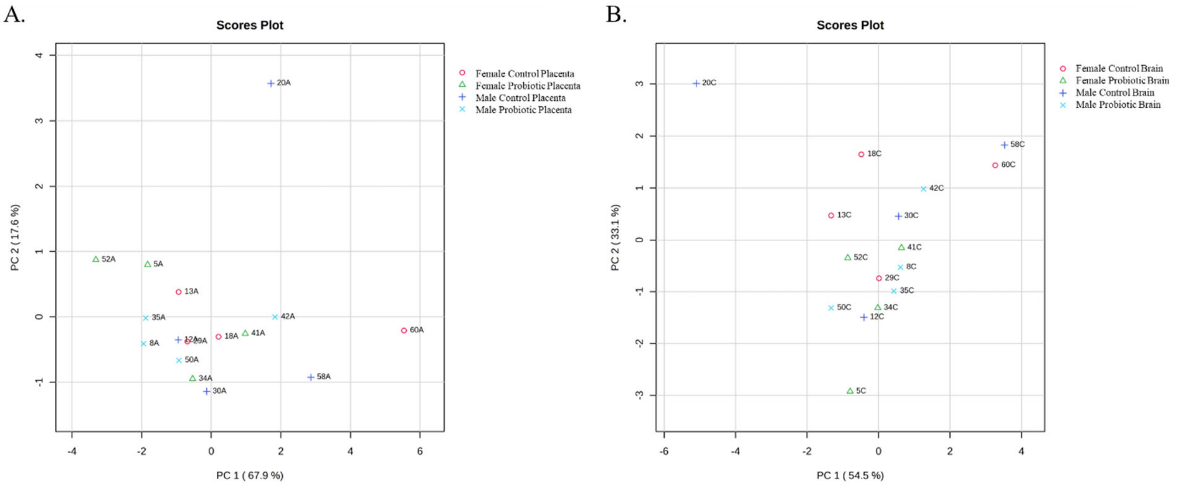

Figure S4. 2D PCA plot of SCFA in placenta and fetal brain samples from probiotic and control dams. A) 2D PCA plot of SCFA in placental samples from these two groups. B) 2D PCA plot of SCFA from fetal brain samples from these two groups. N = 4 replicates/group.

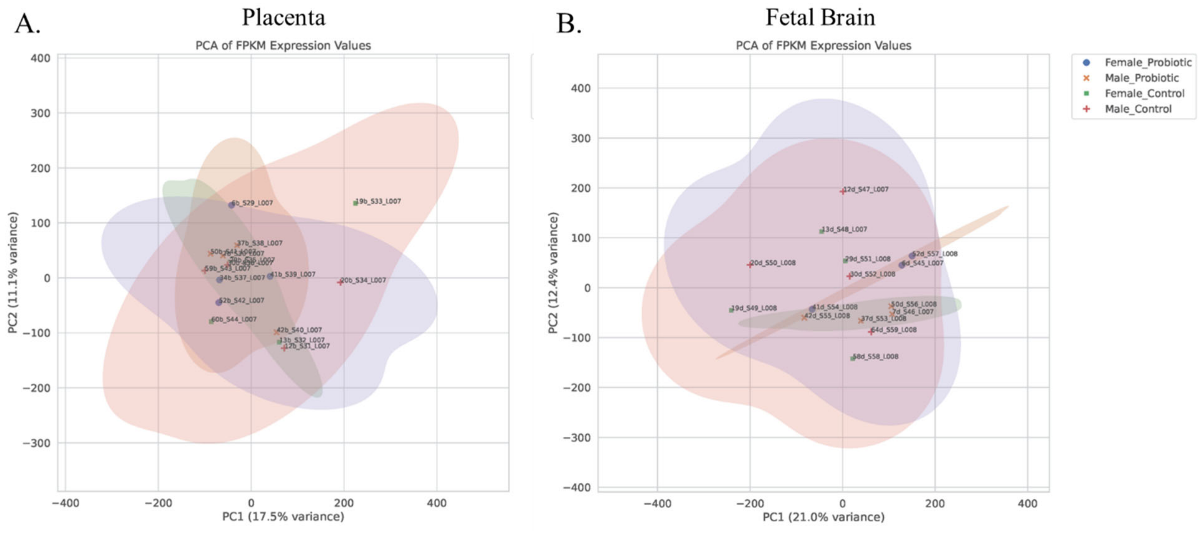

Figure S5. 2D PCA plot of RNAseq results from placenta and fetal brain samples from probiotic and control dams. A) 2D PCA plot of SCFA in placental samples from these two groups. B) 2D PCA plot of SCFA from fetal brain samples from these two groups. No clear separation was evident based on maternal treatment (probiotic vs. control) or sex. PERMANOVA values for placenta and fetal brain were 0.66 and 0.54, respectively. N = 4 replicates/group, other than for female brain samples from probiotic-treated dams that had 3 replicates.

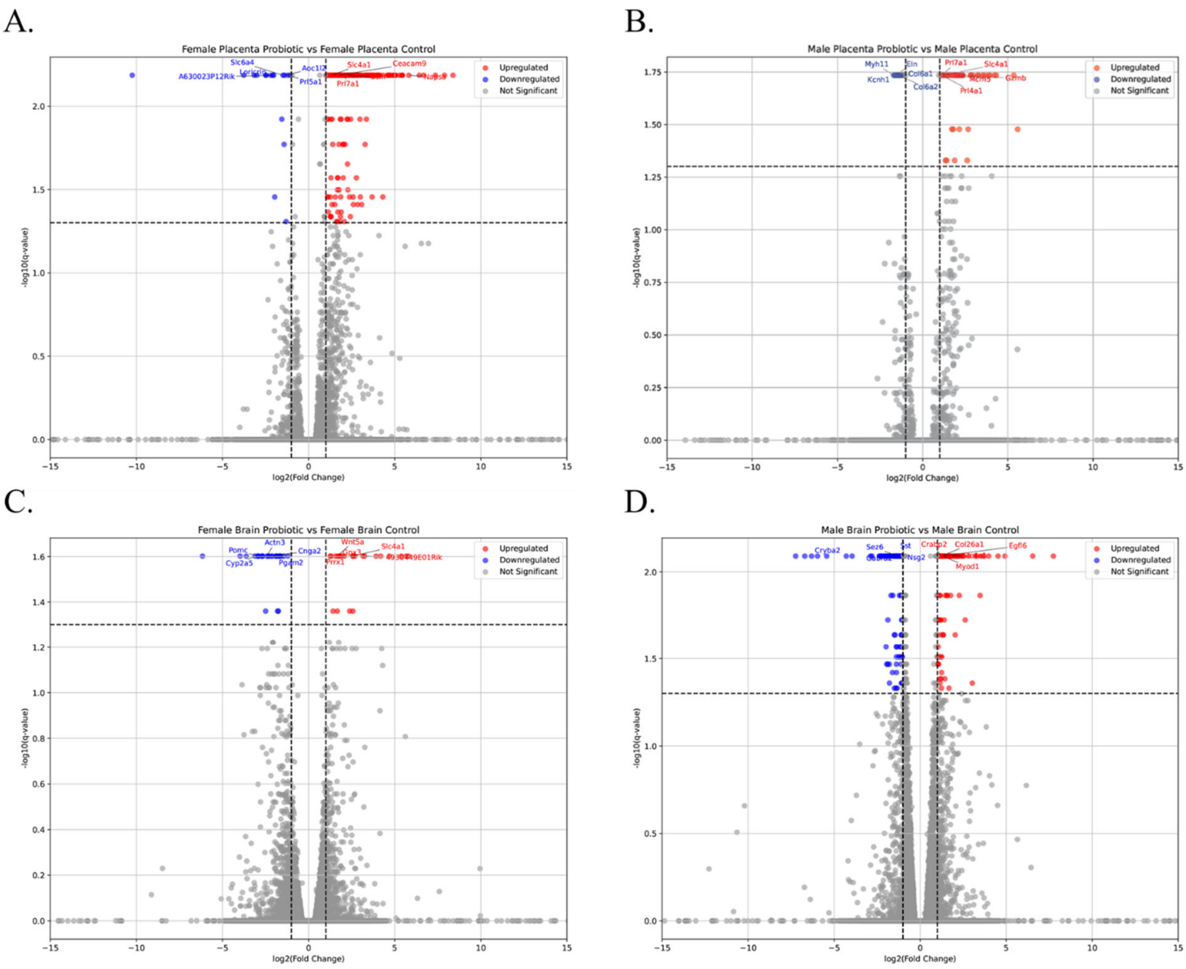

Figure S6. Volcano plots showing differentially expressed genes (DEG) for female and male placenta and fetal brain from maternal probiotic treated vs. counterpart controls. A) Volcano plot comparison of DEG in placenta female placenta derived from probiotic-treated dams vs. female placenta derived from control dams. B) Volcano plot comparison of DEG in placenta male placenta derived from probiotic-treated dams vs. male placenta derived from control dams. C) Volcano plot comparison of DEG in fetal brain of female conceptuses derived from probiotic-treated dams vs. female fetal brain derived from control dams. D) Volcano plot comparison of DEG in fetal brain of male conceptuses derived from probiotic-treated dams vs. male fetal brain derived from control dams.

A. B.

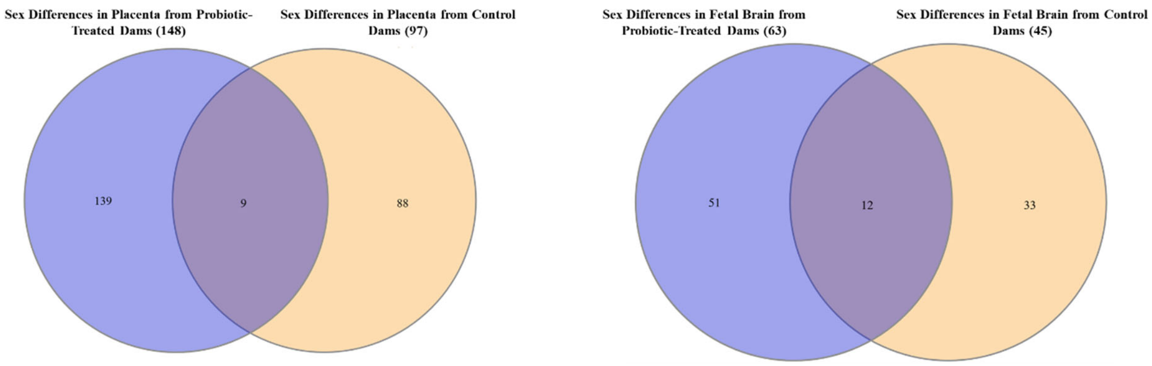

Figure S7. Venn diagram comparisons of sex differences in maternal probiotic-treated and control dams for placenta and fetal brain samples. A) Venn diagram comparison of sex differences in placenta samples from probiotic-treated dams vs. sex differences in placenta samples from control dams. B) Venn diagram comparison of sex differences in fetal brain samples from probiotic-treated dams vs. sex differences in fetal brain samples from control dams.

Table S1. Details on probiotic, Bio-Kult® Advanced, ADM Protexin Ltd; 14 bacterial strains plus supplements, used in current study.

**Collective probiotic information**	**Bacterial species within this probiotic supplement**
140 mg (4 billion colony forming units, CFU)	*Bacillus subtilis PXN 21*, *Bifidobacterium bifidum PXN 23*, *Bifidobacterium breve PXN 25*, *Bifidobacterium infantis PXN 27*, *Bifidobacterium longum PXN 30*, *Lactobacillus delbrueckii ssp. bulgaricus PXN 39*, *Lactobacillus casei PXN 37*, *Lactobacillus plantarum PXN 47*, *Lactobacillus rhamnosus PXN 54*, *Lactobacillus helveticus PXN 45*, *Lactobacillus salivarius PXN 57*, *Lactococcus lactis ssp. lactis PXN 63*, *Streptococcus thermophilus PXN 66*

Table S2. The MRM parameters for each short chained fatty acid (SCFA) examined.

SCFA name	Precursor Ion	Product Ion	Cone Voltage (V)	Collision Energy (eV)	Retention time (min)
**Acetic Acid**	194.1	137.1	34	20	5.52
	194.1	151.8	34	16	
**Propanoic Acid**	208.1	137.1	34	16	7.40
	208.1	151.8	34	16	
**Butyric Acid**	222.1	137.1	36	18	9.43
	222.1	151.8	36	16	
**Iso-Butyric Acid**	222.1	137.1	36	20	9.13
	222.1	151.8	36	16	
**Valeric Acid**	236.1	137.1	36	22	11.62
	236.1	151.8	36	16	
**Isovaleric Acid**	236.1	137.1	40	20	11.21
	236.1	151.8	40	18	
**2-Methyl butyric Acid**	236.1	137.1	38	20	10.93
	236.1	151.8	38	28	
**Isobutoxyacetic acid (IS)**	266.2	137.1	40	20	13.48
	266.1	151.7	40	18	

Table S3. Number of raw reads, mapped reads, and % mapped reads for RNA seq results from placenta and fetal brain from maternal probiotic and control treated mice.

Sample	Maternal Treatment and Offspring Sex	Fetal Organ	Raw Reads	Mapped Reads	% of Mapped Reads
6b	Probiotic Female	Placenta	150088406	147099497	98.0085677
34b	Probiotic Female	Placenta	144010056	141566784	98.30340181
41b	Probiotic Female	Placenta	157983944	155531439	98.44762389
52b	Probiotic Female	Placenta	133162486	130962638	98.3479972
7b	Probiotic Male	Placenta	131603642	129040026	98.05201744
37b	Probiotic Male	Placenta	164142128	161692749	98.50776944
42b	Probiotic Male	Placenta	154926742	152023756	98.1262202
50b	Probiotic Male	Placenta	121057362	119140282	98.4163871
13b	Control Female	Placenta	143983632	140523559	97.59689838
19b	Control Female	Placenta	139535238	136858309	98.08153909
29b	Control Female	Placenta	177990086	174108320	97.81911112
60b	Control Female	Placenta	142791086	139930486	97.99665366
12b	Control Male	Placenta	145545548	142514198	97.91724993
20b	Control Male	Placenta	146831138	143719388	97.88072881
30b	Control Male	Placenta	179601360	176107201	98.0544919
59b	Control Male	Placenta	175816096	172833353	98.30348696
6d	Probiotic Female	Brain	149034952	146370883	98.21245355
41d	Probiotic Female	Brain	154064618	151484238	98.32513134
52d	Probiotic Female	Brain	139024086	136729173	98.34926949
7d	Probiotic Male	Brain	149729240	147163394	98.28634273
37d	Probiotic Male	Brain	178221572	175486050	98.46510051
42d	Probiotic Male	Brain	139591050	137561944	98.54639248
50d	Probiotic Male	Brain	120830048	118806205	98.32504991
13d	Control Female	Brain	141661826	138990371	98.11420262
19d	Control Female	Brain	153851218	150998160	98.14557334
29d	Control Female	Brain	175765298	173180138	98.52919773
58d	Control Female	Brain	163942574	161385205	98.44008244
12d	Control Male	Brain	136148748	133277588	97.89115945
20d	Control Male	Brain	175451850	171541516	97.77127799

30d	Control Male	Brain	198035404	195132200	98.53399749
64d	Control Male	Brain	223653978	220244227	98.47543467
**Average**			**155099206.8**	**152322686.4**	**98.2098423**

Table S4. Results for all metabolites in fecal boli samples from control and probiotic-treated mouse dams. Results are reported in ng/mg of tissue.

**Group**	**2 Methyl butanoic Acid**	**Isovaleric Acid**	**Valeric Acid**	**Isobutyric Acid**	**Butyric Acid**	**Propanoic Acid**	**Acetic Acid**
**Control Female Mice Prior to Receiving Vehicle Control**	2.5 ± 0.4	2.5 ± 0.6	7.0 ± 2.1	7.0 ± 1.9	123.4 ± 23.1	135.1 ± 39.7	1331.5 ± 277.3
**Control Female Mice After Receiving Vehicle Control**	3.6 ± 0.7	3.3 ± 0.5	16.0 ± 3.6	8.3 ± 0.8	425.5 ± 178.5	217.9 ± 55.1	1800.9 ± 509.9
**Female Mice Prior to Receiving Probiotic Treatment**	6.1 ± 1.6	7.3 ± 1.9	19.4 ± 5.2	13.2 ± 3.8	314.5 ± 95.7	115.9 ± 46.8	1582.2 ± 358.1
**Female Mice After Receiving Probiotic Treatment**	3.7 ± 0.8	3.7 ± 0.7	14.9 ± 3.6	9.5 ± 2.6	281.6 ± 70.7	253.5 ± 78.5	1983.1 ± 706.8

Table S5. Results for all metabolites in placenta and fetal brain samples from control and probiotic-treated mouse dams. Results are reported in ng/mg of tissue.

Group	2-Methylbutanoic Acid	Isovaleric Acid (	Valeric Acid	Butyric Acid	Isobutyric Acid	Propanoic Acid	Acetic Acid
**Placenta from Control Dams**	0.15 ± 0.05	0.17 ± 0.03	0.79 ± 0.10	1.51 ± 0.32	0.94 ± 0.20	4.33 ± 0.57	553.31 ± 66.90
**Placenta from Probiotic-Treated Dams**	0.24 ± 0.06	0.23 ± 0.04	1.11 ± 0.28	2.17 ± 0.43	1.22 ± 0.19	5.14 ± 0.75	503.48 ± 44.87
**Fetal Brain from Control Dams**	0.29 ± 0.09	0.16 ± 0.02	0.91 ± 0.13	2.37 ± 0.38	1.1 ± 0.21	4.69 ± 0.46	558.76 ± 98.53
**Fetal Brain from Probiotic-Treated Dams**	0.46 ± 0.08	0.19 ± 0.03	0.57 ± 0.11	2.75 ± 0.43	1.34 ± 0.16	4.16 ± 0.57	462.06 ± 61.50
