# Supplementary figures and images for: Susceptibility of the Placenta and Fetal Brain to Maternal Probiotic Supplementation
Source: Microorganisms. 2026 May 22;14(6):1175. doi: 10.3390/microorganisms14061175 (PMC13303452; doi:10.3390/microorganisms14061175)

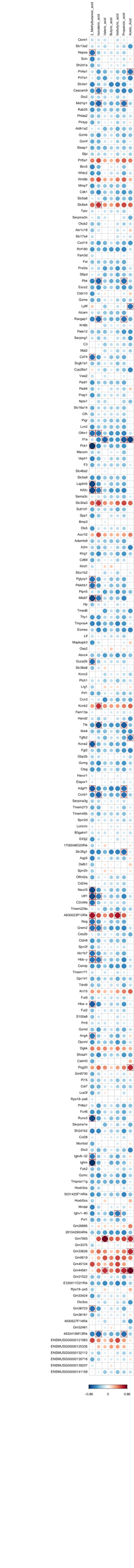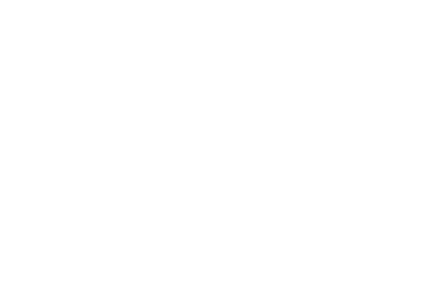

Supplement: Supplementary file 1 [file microorganisms-14-01175-s001.zip › Supplementary File S7_Female Placenta_correlationanalyses.pdf]

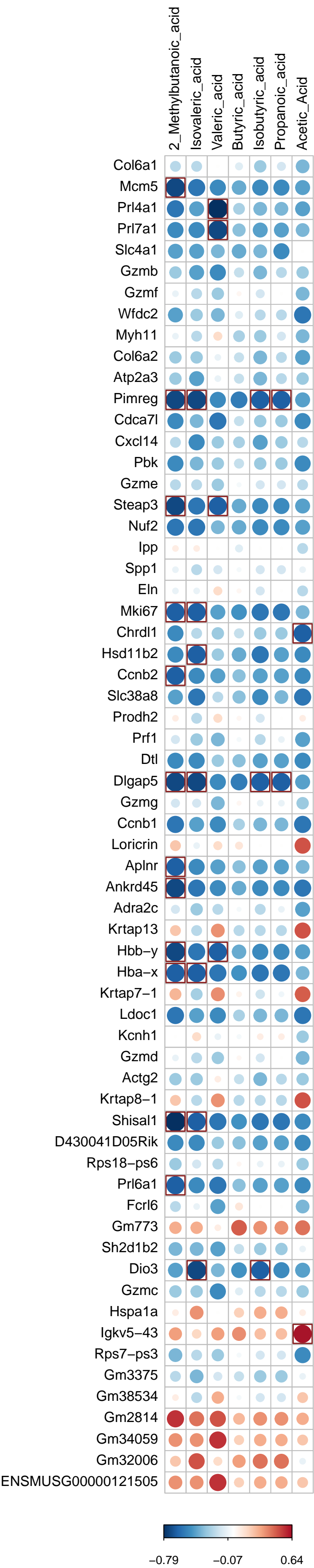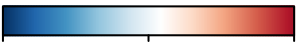

-0.79

-0.07

0.64

Supplement: Supplementary file 1 [file microorganisms-14-01175-s001.zip › Supplementary File S8_Male Placenta_correlationanalyses.pdf]

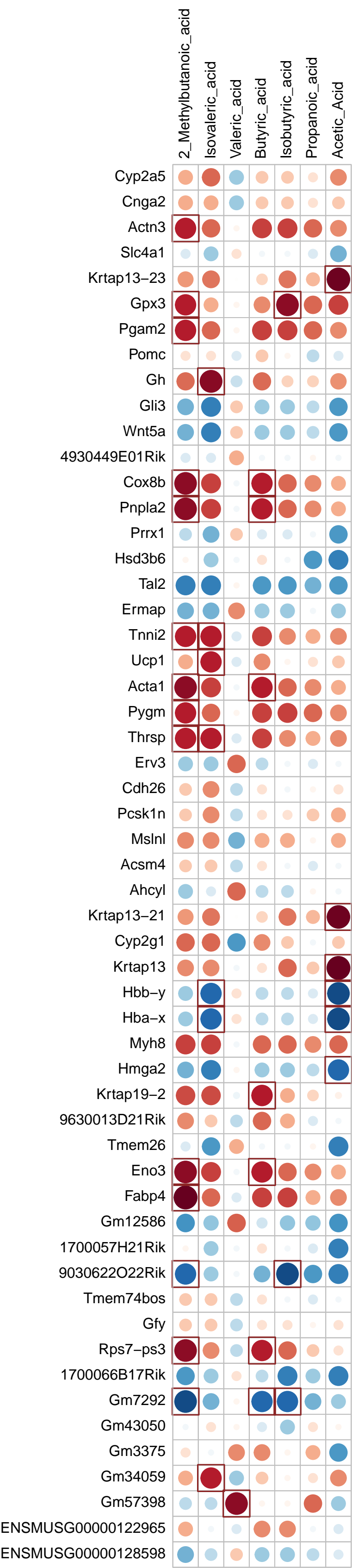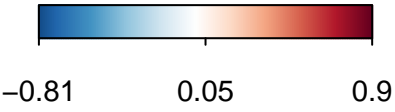

Supplement: Supplementary file 1 [file microorganisms-14-01175-s001.zip › Supplementary File S9_Female Brain_correlationanalyses.pdf]

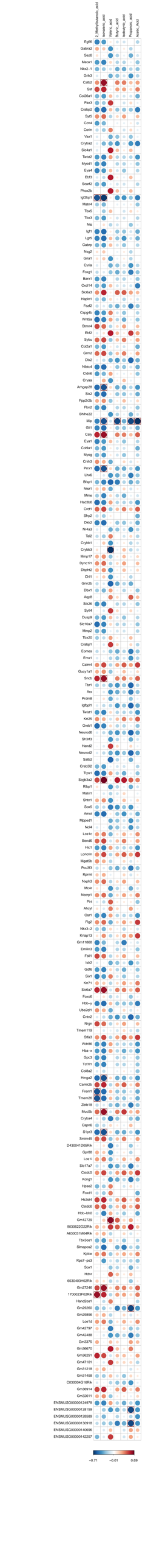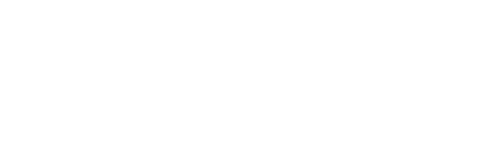

Supplement: Supplementary file 1 [file microorganisms-14-01175-s001.zip › Supplementary File S10_Male Brain_correlationanalyses.pdf]
